# Descriptive study of adverse drug reactions in a tertiary care pediatric hospital in México from 2014 to 2017

**DOI:** 10.1371/journal.pone.0230576

**Published:** 2020-03-24

**Authors:** Olga Morales-Ríos, Carlo Cicero-Oneto, Carlos García-Ruiz, Dina Villanueva-García, Maribelle Hernández-Hernández, Víctor Olivar-López, Rodolfo Norberto Jiménez-Juárez, Luis Jasso-Gutiérrez

**Affiliations:** 1 Clinical Research Department, Hospital Infantil de México Federico Gómez, Ciudad de México, México; 2 Hematological Oncology Department, Hospital Infantil de México Federico Gómez, Ciudad de México, México; 3 Neonatology Department, Hospital Infantil de México Federico Gómez, Ciudad de México, México; 4 Intensive Therapy Department, Hospital Infantil de México Federico Gómez, Ciudad de México, México; 5 Emergency Department, Hospital Infantil de México Federico Gómez, Ciudad de México, México; 6 Infectious Diseases Department, Hospital Infantil de México Federico Gómez, Ciudad de México, México; 7 Department of Pediatrics, Centro Médico Nacional La Raza, Infectious Diseases Hospital, Instituto Mexicano del Seguro Social, Ciudad de México, México; National Chiao Tung University College of Biological Science and Technology, TAIWAN

## Abstract

**Introduction:**

In Pediatrics, adverse drug reactions (ADRs) affect morbidity and mortality. In Mexico, the characteristics of ADRs and suspect drugs have not been described in hospitalized children.

**Objective:**

To estimate the frequency of ADRs and describe them, as well as suspect drugs, in a tertiary care pediatric hospital in Mexico.

**Methods:**

A total of 1,649 Hospital Infantil de Mexico Federico Gómez ADR reports were analyzed. Completeness of the information was assessed, and ADRs severity and seriousness were assigned based on NOM-220-SSA1-2012, with causality being established according to the Naranjo algorithm. ADRs were classified with WHO Adverse Drug Reaction Terminology (WHO-ART). The drugs involved in ADRs were categorized according to the Anatomical Therapeutic Chemical (ATC) classification. Descriptive analysis was performed using the SPSS 20 statistical package.

**Results:**

Of all the reports, 5.8% lacked sufficient information for the analysis (grade 0). ADRs frequency ranged from 2.12% to 8.07%. ADRs occurred most commonly in children (56.9%), in the female gender (52%), in subjects with normal BMI Z-score (46.6%) and malnutrition (35.3%), diagnosed with neoplasms (72.2%) and in the Emergency Department (70.0%). ADRs were severe in 14.4% of cases, in 81.0% they were serious and 2.1% were classified as definite. Most common serious ADR was febrile neutropenia (44.5%). The 0.7% of patients recovering with sequelae; 1.1% died (with the medication being associated) and 70.3% were admitted to the hospital as a result of an ADR. Antineoplastic and immunomodulating agents were more commonly associated with serious ADRs.

**Conclusion:**

ADRs affected morbidity and mortality, which is why strengthening pharmacovigilance programs in Mexican pediatric hospitals is necessary.

## Introduction

Adverse drug reactions (ADRs) have been defined by the World Health Organization (WHO) as “a response to a drug which is noxious and unintended, and which occurs at doses normally used in man for the prophylaxis, diagnosis, or therapy of disease, or for modification of physiological function” [1]. In children, ADRs surveillance and documentation is crucial to monitor the safe use of the medications used in this population since, compared to adults, children may have higher vulnerability for experiencing ADRs because: **a)** clinical trials carried out in these population are scarce [2,3]; **b)** unlicensed and off-label drugs are often prescribed [4]; **c)** there is the possibility of exposure to medications during prenatal stage and breastfeeding [5], **d)** children may react differently than adults to administered medications, which can be explained by changes in absorption, distribution, metabolism and excretion [6], and **e)** hospitalized pediatric patients can be administered more than 10 drugs during their stay [7,8]. In the pediatrics setting, ADRs generate a significant impact on morbidity, mortality [9–11] and costs [12,13]. Frequency in hospitalized children ranges from 1.5% to 18.1%, while in outpatients it ranges from 0.7% to 2.74%; in addition 0.2% to 5.8% result in hospitalization [14,15]. On the other hand, 7.7% of reports received by the Uppsala Monitoring Center involve children [16].

In 1999, Mexico joined the WHO Programme for International Drug Monitoring [17] and, ever since, the National Pharmacovigilance Center (CNF–Centro Nacional de Farmacovigilancia) is responsible for coordinating pharmacovigilance activities in the country, supported by an organization in which the Institutional Pharmacovigilance Centers (CIF–Centros Institucionales de Farmacovigilancia), the State Pharmacovigilance Centers and the Pharmaceutical Industry participate [18]. However, in spite of the above, studies in children are limited in Mexico [19–23] and there are no works reporting ADRs frequency and characteristics, as well as associated suspect medications, in pediatric hospital patients.

### Aim of the study

To estimate the frequency and describe the characteristics (severity, seriousness, causality, management and outcomes) of ADRs occurred in Hospital Infantil de Mexico Federico Gómez (HIMFG) hospitalized patients, as well as to describe associated suspect drugs.

## Methods

### Study design

This study is a secondary analysis of the HIMFG Electronic Pharmacovigilance Program database from January 2014 to December 2017.

### Study population and settings

The study was conducted at HIMFG, which is a tertiary care pediatric hospital in Mexico with 229 registered beds. Hospital discharges during the study period ranged from 7,369 to 7,920 [24]. The HIMFG CIF designed the Electronic Pharmacovigilance Program for online ADR report filing from different hospitalization areas [25]. From January 2014 to December 2017, the Electronic Pharmacovigilance Program received 2,099 ADR reports, out of which 348 (16.6%) were excluded for belonging to a research protocol and one (0.05%) was a vaccine report.

### Database and data collection

The data collected in the Electronic Pharmacovigilance Program included patient demographics, ADRs description, information on the suspect drug, information on concomitant pharmacotherapy, patient medical history and data of the personnel that generated the report [25]. The ADR reports were received at the HIMFG CIF, where a pharmacist analyzed them according to the Official Mexican Standard NOM-220-SSA1-2012, “Pharmacovigilance Implementation and Operation” [26]. A search for repeated cases was carried out using Microsoft Excel 2013 duplicate value identification tool; however, it is possible that some duplicate values were not detected. All reports were electronically submitted to the CNF using the SISCE v1.2.2014 database.

### ADR reports quality

The criteria to classify the completeness of the information contained in ADR reports was defined as [26]:

- Grade 0. When the report includes an identifiable patient, a suspected ADR and data on the reporter.
- Grade 1. When in addition to Grade 0 data, the date of ADR onset, as well as the dates of suspect drug initiation and discontinuation are included.
- Grade 2. When in addition to Grade 1 data, the name of the drug substance, the commercial name, dosage, route of administration and the reason for prescription of the suspect drug, as well as consequences of the event and medical history relevant data are included.
- Grade 3. When in addition to Grade 2 data, the reappearance of the clinical manifestation resulting from suspect drug re-administration is included (re-administration-positive).

### ADRs frequency

To estimate ADRs overall frequency, the total number of patients with ADRs was divided by the total number of hospital discharges during the study period and the result was multiplied by 100.

### Adverse drug reactions (ADRs) characteristics

ADRs were defined according to the World Health Organization and the Official Mexican Standard NOM-220-SSA1-2012, “Pharmacovigilance Implementation and Operation” [1,26]. ADRs were classified according to the WHO Adverse Drug Reaction Terminology (WHO-ART) catalog, which was integrated in the SISCE v1.2.2014 database. Each report was assessed for ADRs severity, seriousness and causality.

The ADRs severity classification was modified based on NOM-220-SSA1-2012 [26] as follows:

- Mild: ADRs not requiring pharmacological treatment or suspect drug discontinuation.
- Moderate: ADRs that require pharmacological treatment and no suspect drug discontinuation.
- Severe: ADRs that require both pharmacological treatment and suspect drug discontinuation.

The seriousness was classified as [26]:

- Serious ADRs: ADRs that: a) Result in patient death, b) Put patient’s life in danger at the very moment they occur, c) Require hospitalization or prolongation of existing hospitalization, d) Result in persistent or significant disability or incapacity and e) Are the result of congenital anomalies or birth defects.
- Non-serious: ADRs not meeting the above-specified criteria.

Causality of each report was assigned using Naranjo’s algorithm [27] as:

- Definite (≥ 9 points)
- Probable (5–8 points)
- Possible (1–4 puntos)
- Doubtful (≤ 0)

### Management and outcomes

Patient management was reported as: **a)** ADR resulted in hospital admission, **b)** Medication was stopped because of ADRs, and **c)** ADR required treatment. Patient outcomes were reported as: **a)** Recovered without sequels, **b)** Recovered with sequels, **c)** Death (drug-related), **d)** Death (not drug-related) and **e)** Unknown (not documented in the report).

### Drugs involved in ADRs

The drugs involved in ADRs were categorized into various drug classes according to the Anatomical Therapeutic Chemical (ATC) classification, based on the 2018 WHO-ATC Index [28].

### Data analyses

For descriptive purposes, patients were classified into 4 categories according to the body mass index (BMI) Z-score (normal weight, obesity, overweight and malnutrition) [29] and 3 categories according to age (infants, children and adolescents) [30]. The International Statistical Classification of Diseases, 10^th^ Revision (ICD 10), was used for all diagnoses [31]. The descriptive analyses included an estimation of relative frequencies for qualitative variables such as number of cases and percentages. The intra-group difference for categorical variables (age group, gender, body mass index category according to Z-score, type of diagnoses and location during the reaction) was calculated using the chi-square test. In all cases, a p-value < 0.05 was considered to be statistically significant. Statistical analysis was performed using the SPSS 20 statistical package.

### Ethical approval

The study was approved by the Hospital Infantil de Mexico Federico Gómez Research Commission, Ethics and Biosafety Committees with authorization number HIM 2018–003 SSA 1470. Patient confidentiality was protected in the SISCE v1.2.2014 database. Patient medical records were not reviewed in this research protocol. It is important mentioning that no information identifying the patients, such as names, initials or institutional registry number, will be disclosed in the publication or the available restriction-free database.

## Results

### ADR reports quality

A total of 1,750 ADR reports were reviewed, out of which 308 (17.6%) were grade 3, 1,341 (76.6%) grade 2 and 101 (5.8%) grade 0. Grade 0 reports were excluded because they did not have enough information for the analysis, and the results of this work are therefore based on 1,649 patient reports that contained 2,166 ADR clinical manifestations.

### ADR frequency

The frequency of ADRs in 2014 was 8.07%, in 2015 it was 5.06%, in 2016 it was 6.52% and in 2017 it was 2.12% **(S1 Fig)**.

### Patient demographics

The age group with the highest ADR frequency was the group of children (2 to 11 years), with 56.9% (p < 0.05). The female gender had an ADR frequency of 52%, whereas in males it was 48% (*p* > 0.05). The highest ADR frequency was observed in patients with normal weight (46.6%), followed by patients with malnutrition (35.3%) (p < 0.05). The main diagnoses were neoplasms (C00-D48) with 72.2%, followed by congenital malformations, deformities and chromosomal abnormalities (Q00-Q99) with 10.1% (p < 0.05). The reports were mainly from Emergency Department patients (70.0%) (p < 0.05) (Table 1).

**Table 1:** Patient demographics.

	Total number of patients n = 1,649 n (%)	*p* value
**Age**		
Neonates (0 to 27 days)	64 (3.9)	0.000*
Infants and toddlers (28 days to 23 months)	224 (13.6)	
Children (2 to 11 years)	939 (56.9)	
Adolescents (12 to 18 years)	384 (23.3)	
Adults (≥ 18 years)	16 (1)	
No data	22 (1.3)	
**Gender**		
Females	857 (52)	0.109
Males	792 (48)	
**BMI Z-score**		
Obesity	117 (7.1)	0.000*
Overweight	159 (9.7)	
Normal weight	769 (46.6)	
Malnutrition	582 (35.3)	
No data	22 (1.3)	
**Diagnoses**		
C00-D48 Neoplasms	1190 (72.2)	0.000*
Q00-Q99 Congenital malformations, deformations and chromosomal abnormalities	167 (10.1)	
J00-J99 Diseases of the respiratory system	46 (2.8)	
D50-D89 Diseases of the blood	45 (2.7)	
K00-K93 Diseases of the digestive system	29 (1.8)	
Z00-Z99 Factors influencing health status and contact with health services	24 (1.5)	
I00-I99 Diseases of the circulatory system	23 (1.4)	
N00-N99 Diseases of the genitourinary system	22 (1.3)	
G00-G99 Diseases of the nervous system	21 (1.3)	
A00-B99 Certain infectious and parasitic diseases	18 (1.1)	
Others^a^	49 (2.9)	
No data	15 (0.9)	
**Location during reaction**		
Emergency Department	1153 (70.0)	0.000*
Surgical Intensive Therapy Unit	178 (10.8)	
Pediatric Intensive Therapy Unit	131 (7.9)	
Neonatology Department	84 (5.0)	
Infectious Diseases Department	41 (2.5)	
Nephrology Department	31 (1.9)	
Others^b^	31 (1.9)	

^a^ Others: P00-P96 Certain conditions originating in the perinatal period, E00-E90 Endocrine, nutritional and metabolic diseases, M00-M99 Diseases of the musculoskeletal system and connective tissue, R00-R99 Symptoms, signs and abnormal clinical and laboratory findings, not elsewhere classified, F00-F99 Mental and behavioral disorders, S00-T98 Injury, poisoning and certain other consequences of external causes.

^b^Others: Adolescents Department, Gastroenterology Department, Anesthesiology Department, General Surgery Department, Allergy Department, Internal Medicine Department, Dermatology Department

*p < 0.05

### Adverse drug reactions (ADRs) characteristics

Among total reported ADRs, 14.4% were severe and 81.0% were serious. On the other hand, 58.6% of ADRs were probable, 38.9% possible, 2.1% definite and 0.4% doubtful. Patients not diagnosed with Neoplasms (C00-D48) had 14.9% of severe ADRs, 51.9% of non-serious ADRs y 78% of probable ADRs, whereas those diagnosed with Neoplasms (C00-D48) had 14.2% of severe ADRs, 95.7% of serious ADRs and 49.9% of probable ADRs (Table 2).

**Table 2:** Severity, seriousness and causality of total ADRs, ADRs in patients not diagnosed with Neoplasms and in patients diagnosed with Neoplasms.

	Total ADRs n = 2,166 n (%)	ADRs in patients without Neoplasms (C00-D48) n = 672 n (%)	ADRs in patients with Neoplasms (C00-D48) n = 1,494 n (%)
**Severity**		** **	
Severe	312 (14.4)	100 (14.9)	212 (14.2)
Moderate	1,699 (78.4)	433 (64.4)	1,266 (84.7)
Mild	155 (7.2)	139 (20.7)	16 (1.1)
**Seriousness**		** **	
Serious	1,753 (81.0)	323 (48.1)	1,430 (95.7)
Non-serious	413 (19.0)	349 (51.9)	64 (4.3)
**Causality**		** **	
Definite	45 (2.1)	23 (3.4)	22 (1.5)
Probable	1,270 (58.6)	524 (78.0)	746 (49.9)
Possible	842 (38.9)	122 (18.2)	720 (48.2)
Doubtful	9 (0.4)	3 (0.4)	6 (0.4)

The most common serious ADR was febrile neutropenia (52.4%) in patients diagnosed with Neoplasms (C00-D48) (Table 3), while the most common non-serious ADR in patients not diagnosed with Neoplasms (C00-D48) was abnormal electrolytes (26.6%), (Table 4).

**Table 3:** 10 most common serious ADRs in all patients, in patients not diagnosed with Neoplasms and in patients diagnosed with Neoplasms.

	Serious ADRs in all patients n = 1,753 n (%)	Serious ADRs in patients without Neoplasms (C00-D48) n = 323 n (%)	Serious ADRs in patients with Neoplasms (C00-D48) n = 1,430 n (%)
Febrile neutropenia	780 (44.5)	31 (9.6)	749 (52.4)
Sepsis	147 (8.4)	7 (2.2)	140 (9.8)
Abnormal electrolytes^a^	134 (7.6)	99 (30.7)	35 (2.4)
Septic shock	101 (5.8)	1 (0.3)	100 (7.0)
Pancytopenia	98 (5.6)	4 (1.2)	94 (6.6)
Mucositis	45 (2.6)	2 (0.6)	43 (3.0)
Pancreatitis	27 (1.5)	5 (1.5)	22 (1.5)
Thrombocytopenia	23 (1.3)	4 (1.2)	19 (1.3)
Hypotension	21 (1.2)	15 (4.6)	6 (0.4)
Neutropenic colitis	16 (0.9)	1 (0.3)	15 (1.0)
Others	361 (20.6)	154 (47.7)	207 (14.5)

^a^Abnormal electrolytes: Calcium deficiency, calcium depletion, decreased blood chloride, hyperphosphatemia, hypernatremia, hyperkalemia, hypocalcemia, hypochloremia, hypophosphatemia, hypokalemia, hypomagnesemia, hyponatremia, decreased serum potassium, increased serum potassium, decreased plasma sodium.

**Table 4:** 10 most common non-serious ADRs in all patients, in patients not diagnosed with Neoplasms and in patients diagnosed with Neoplasms.

	Non-Serious ADRs in all patients n = 413 n (%)	Non-Serious ADRs in patients without Neoplasms (C00-D48) n = 349 n (%)	Non-Serious ADRs in patients with Neoplasms (C00-D48) n = 64 n (%)
Abnormal electrolytes^a^	108 (26.2)	93 (26.6)	15 (23.4)
Cutaneous eruption^b^	31 (7.5)	22 (6.3)	9 (14.1)
Hyperglycemia	30 (7.3)	27 (7.7)	3 (4.7)
Tachycardia	22 (5.3)	20 (5.7)	2 (3.1)
Increased aspartate aminotransferase	14 (3.4)	13 (3.7)	1 (1.6)
Increased alanine aminotransferase	12 (2.9)	11 (3.2)	1 (1.6)
Vomiting	12 (2.9)	11 (3.2)	1 (1.6)
Fever	10 (2.4)	8 (2.3)	2 (3.1)
Hypotension	10 (2.4)	9 (2.6)	1 (1.6)
Nausea	10 (2.4)	8 (2.3)	2 (3.1)
Others	154 (37.3)	127 (36.4)	27 (42.1)

^a^Abnormal electrolytes: Calcium deficiency, calcium depletion, decreased blood chloride, hyperphosphatemia, hypernatremia, hyperkalemia, hypocalcemia, hypochloremia, hypophosphatemia, hypokalemia, hypomagnesemia, hyponatremia, decreased serum potassium, increased serum potassium, decreased plasma sodium.

^b^Cutaneous eruption: erythema, rash, urticaria, exanthema, erythema multiforme, pruritus.

### Management and outcomes

In 70.3% of total patients, ADRs were the cause of hospital admission, 15.2% of the patients required the withdrawal of the suspect drug and 83.6% required treatment for ADRs. Recovery from the ADRs without sequelae was observed in 90.8% of patients; however 0.7% recovered with some sequel. On the other hand, in 1.1% of patients, death was related to the suspect drug. Among the patients diagnosed with Neoplasms (C00-D48), 91.9% required hospital admission due to ADRs, 47.3% of the patients not diagnosed with Neoplasms (C00-D48) required the withdrawal of the suspect drug, whereas 94.5% of patients with Neoplasms (C00-D48) required treatment for ADRs. Of the patients without Neoplasms (C00-D48), 1.1% recovered from the ADR with some sequel. On the other hand, in 1.4% of patients with Neoplasms (C00-D48), death was related to the suspect drug (Table 5).

**Table 5:** ADRs management and outcomes in all patients, in patients not diagnosed with Neoplasms (C00-D48) and in patients diagnosed with Neoplasms (C00-D48).

	Total number of patients n = 1,649 n (%)	Patients without Neoplasms (C00-D48) n = 444 n (%)	Patients with Neoplasms (C00-D48) n = 1,190 n (%)	Undiagnosed patients n = 15 n (%)
**ADRs resulting in hospital admission**				
Yes	1,160 (70.3)	58 (13.1)	1,094 (91.9)	8 (53.3)
No	489 (29.7)	386 (86.9)	96 (8.1)	7 (46.7)
**Stopped the medication**				
Yes	250 (15.2)	210 (47.3)	36 (3.0)	4 (26.7)
No	1,399 (84.8)	234 (52.7)	1,154 (97.0)	11 (73.3)
**ADRs requiring treatment**				
Yes	1,378 (83.6)	244 (55.0)	1,124 (94.5)	10 (66.7)
No	271 (16.4)	200 (45.0)	66 (5.5)	5 (33.3)
**Outcomes**				
Recovered with no sequel	1,498 (90.8)	348 (78.4)	1,136 (95.5)	14 (93.3)
Recovered with sequel	11 (0.7)	5 (1.1)	6 (0.5)	0 (0.0)
Death–drug-related	18 (1.1)	1 (0.22)	17 (1.4)	0 (0.0)
Death–not drug-related	5 (0.3)	5 (1.1)	0 (0.0)	0 (0.0)
Unknown	117 (7.1)	85 (19.2)	31 (2.6)	1 (6.7)

Kidney failure was the most common sequel in those patients who recovered with any (Table 6). Febrile neutropenia was the ADR that most often occurred in patients whose death was related to the suspect drug (Table 7).

**Table 6:** Patients with “Recovered with sequel” outcome.

Patient data	Diagnoses	Sequel-associated ADR	Sequel	Suspect drug	Concomitant drugs
Female 15 years, 1 month	Nasopharyngeal carcinoma	Acute kidney failure	Acute kidney failure	Cisplatin	None
Female 12 years, 10 months	Acute lymphoblastic leukemia	Acute kidney failure, increased hepatic enzymes	Acute kidney failure	Methotrexate	None
Female 17 years, 6 months	Serious mitral insufficiency	Serum electrolyte decrease (potassium, sodium, chloride), metabolic alkalosis, acute kidney failure (probable tubular necrosis), oliguria-anuria	Acute kidney failure	Furosemide	None
Female 7 months	Heart failure	Serum electrolyte decrease (potassium), acute kidney failure (tubular necrosis)	Acute kidney failure	Furosemide	Spironolactone
Male 12 years, 10 months	Acute myeloid leukemia	Neutropenic colitis	Hemicolectomy	Cytarabine	Etoposide, mercaptopurine, doxorubicin
Male 1 year, 2 months	Acute lymphoblastic leukemia	Febrile neutropenia, septic shock with abdominal focus	Total colectomy	Cytarabine	Etoposide
Male 3 years, 1 month	Acute lymphoblastic leukemia	Lumbosacral area increased volume, abdominal pain	Lumbosacral region graft	Asparaginase	Dexamethasone, Vincristine,
Female 3 years, 5 months	Biliary duct atresia, chicken pox	Gastrointestinal bleeding	Varix ligation	Ibuprofen	Acyclovir, propranolol, prednisone
Female 2 months	Anorectal malformation with fistula	Low urinary output	Nephrocalcinosis	Furosemide	Domperidone, spironolactone
Female 13 years, 7 months	End-stage chronic renal disease	Hyperglycemia	Diabetes mellitus	Methylprednisolone	None
Male 11 years, 6 months	Right eye retinoblastoma	End-stage chronic kidney failure	End-stage chronic kidney failure	Cisplatin	Cyclophosphamide, Vincristine

**Table 7:** Patients with the “Death–drug-related” outcome.

Patient data	Diagnosis	ADR associated with patient death	Suspect drug	Concomitant drugs
Male 5 years, 2 months	Acute lymphoblastic leukemia	Febrile neutropenia, septic shock	Cytarabine	Fludarabine, idarubicin
Female 2 years, 10 months	Acute lymphoblastic leukemia	Febrile neutropenia, septic shock	Methotrexate	None
Female 17 years, 6 months	Hodgkin’s lymphoma	Febrile neutropenia, septic shock ** Serum creatinine increase that brought the patient to hemodialysis	Methotrexate	None
Female 15 years, 1 month	Acute lymphoblastic leukemia	Febrile neutropenia, pancreatitis	Asparaginase	Cyclophosphamide, Daunorubicin, Vincristine, Dexamethasone
Female 8 years, 2 months	Acute lymphoblastic leukemia	Febrile neutropenia, pancreatitis	Asparaginase	Daunorubicin, Vincristine
Male 14 years, 6 months	Medulloblastoma	Neutropenic colitis, sepsis	Vincristine	Irinotecan, temozolomide
Female 10 years, 3 months	Acute lymphoblastic leukemia	Diabetes Mellitus, febrile neutropenia, septic shock	Vincristine	Daunorubicin, asparaginase
Female 11 years, 6 months	Osteosarcoma	Febrile neutropenia, septic shock	Methotrexate	None
Male No “age” data	Acute lymphoblastic leukemia	Febrile neutropenia, septic shock	Cytarabine	Etoposide, daunorubicin
Male 15 years, 6 months	Acute lymphoblastic leukemia	Febrile neutropenia, septic shock	Cytarabine	Methotrexate
Male 4 years, 11 months	Hepatoblastoma	Febrile neutropenia, septic shock	Doxorubicin	Cisplatin, fluorouracil, Vincristine
Female 16 years, 1 month	Acute lymphoblastic leukemia	Mucositis, pancytopenia, septic shock, Stevens Johnson syndrome	Methotrexate	Cytarabine
Male 18 years, 10 months	Acute lymphoblastic leukemia	Febrile neutropenia, septic shock	Methotrexate	None
Female 15 years, 4 months	Rhabdomyosarcoma	Diarrhea, febrile neutropenia, septic shock	Irinotecan	Doxorubicin, cisplatin
Female 7 years, 4 months	Hodgkin’s lymphoma	Febrile neutropenia, decompensated mixed shock, pulmonary infection that required lung resection, multiple organ failure	Ifosfamide	Methotrexate, Etoposide, Dexamethasone
Female 13 years, 9 months	Acute myeloid leukemia	Febrile neutropenia, hemorrhage	Tretinoin	None
Male 3 years, 5 months	Acute lymphoblastic leukemia	Chicken pox	Vincristine	Daunorubicin, Asparaginase, Dexamethasone
Male 2 years, 4 months	Ventricular septal defect	Pulmonary hemorrhage	Heparin	None

Febrile neutropenia was the ADR that most resulted in hospital admissions (Table 8).

**Table 8:** Main ADRs resulting in hospital admission.

	n (%)
Febrile neutropenia	774 (53.7%)
Sepsis	190 (13.2%)
Pancytopenia	97 (6.7%)
Septic shock	57 (4.0%)
Mucositis	45 (3.1%)

### Drugs involved in ADRs

The drugs that more often were involved in serious ADRs were group L medications (Antineoplastic and immunomodulating agents), with 81.2% (S1 Table), while the drugs that were most commonly related to non-serious ADRs were group C drugs (Cardiovascular system) with 34.9% (S2 Table).

## Discussion

To the best of our knowledge, the present study is the only one that has assessed ADRs causality, type, severity, seriousness and quality of ADR reports information in children from a tertiary care pediatric hospital in Mexico [14,15,32–35]. The ADR reports received at the CIF had sufficient information to carry out the analysis of this work, since 94.2% were grade 2 and grade 3, which was expected, since, in Mexico, hospitals are generators of the ADR reports with the best quality of information [36].

ADRs estimated frequency in the present study ranges from 2.12% to 8.07%, which is consistent with that reported in the literature [14,15,35]; however, these figures should be interpreted with caution, since: **a)** although doctors identify ADRs, they do not report them [37], which causes an underreporting of 93.9% [38] and, therefore, the magnitude of the problem is underestimated, and **b)** the methodologies used in the studies are different, which makes comparison between them difficult. On the other hand, variations in ADR frequency could be attributed to factors such as: **a)** under-reporting [38], since ADRs notification at HIMFG is voluntary; **b)** the CIF temporarily assigned pharmacy students to hospitalization areas in order for them to take care of reporting the ADRs experienced by patients, given that, at HIMFG, there are no clinical pharmacists who, among other things, are qualified to report ADRs; and **c)** one of the authors of this study (CCO) implemented, among the activities of the hematological oncology department residents, the report of ADRs experienced by patients diagnosed with any neoplastic disease.

The fact that ADRs occurred more often in the group of older patients (2 to 11 years and 12 to 18 years), in the female gender and in subjects diagnosed with neoplasms (C00-D48) can be explained by the fact that some of these variables have been confirmed as risk factors for ADRs in children [34,39,40], while malnutrition has also been associated with a high rate of ADRs in children diagnosed with neoplasms [41]. It is important mentioning that none of the above variables can be interpreted or be inferred as risk factors in the study population, since, due to its nature, the study has not that scope. ADRs occurred mainly in the Emergency Department, which could be attributed to the febrile neutropenia reaction induced by medications administered to patients with cancer diagnoses [42].

Severity (mild, moderate, severe) describes the intensity of a clinical manifestation in the patient, whereas seriousness is based on the outcome of the event or the associated action to treat it [1,26]. It is difficult to compare the results of this work with others published in terms of severity, seriousness and causality, since ADRs classifications employed in the literature show considerable heterogeneity [33,34]. In this study, the NOM-220-SSA1-2012 classification of severity was modified because it is ambiguous and generated uncertainty in the assessment. For example, to classify ADRs as moderate and severe, the parameter of requiring or prolonging hospitalization due to an ADR is not considered, while in mild reactions, the fact that hospitalization is not required or prolonged is explicitly mentioned. In addition, it does not describe where to include ADRs that only require suspect drug discontinuation and no treatment. To facilitate the comparison of results with other studies, it is important for NOM-220 future versions to be revised and for other more objective scales used by other authors to assess severity, such as the Hartwig SC et al. scale, to be included [43]. NOM-220 uses the definition of seriousness of the International Conference on Harmonization [1] that has already been used by other authors [44]. In pharmacovigilance, the measurement of causality is essential to establish whether there is a causal relationship between any clinical manifestation and a medication, and to assess it, different algorithms have therefore been proposed [45]. In this work, causality was determined with the Naranjo algorithm [27], since it is the most widely used in pediatric studies [34] and because it is less prone to subjective variations [46]; however, there are authors who suggest that the Naranjo algorithm is not a consistent tool for the assessment of ADRs in hospital environments [47]. Most ADRs were probable and possible, which could be explained by the fact that 44.5% of ADRs were of oncological origin (febrile neutropenia) and, to analyze them with the Naranjo algorithm, it is necessary to consider: **a)** that in chemotherapy regimens, other drugs are administered that can also explain the observed ADRs as, for example, febrile neutropenia and **b)** it cannot be assessed whether ADRs improve when the treatment is interrupted, since the manifestations occur days after chemotherapy is administered as, for example, febrile neutropenia. On the other hand, in most cases it was not feasible to assess re-administration of the drug due to the limitations inherent to treatments and ethical constraints, which explains that only 2.1% of ADRs were definite. In this work, ADRs were described by seriousness because NOM-220-SSA1-2012 uses this criterion to establish the regulatory obligations of participants in pharmacovigilance activities in Mexico [26].

The most common serious ADRs were febrile neutropenia and sepsis, which can be explained by the fact that **a)** they are the most commonly occurring ADRs because patients who are mainly treated at HIMFG have diagnoses of oncological nature [24] or **b)** these are the reactions doctors reported more frequently to the CIF owing to their clinical relevance. Abnormal electrolytes was the non-serious ADR that occurred most often, which can largely be explained by the fact that it is the ADR most frequently attributed to furosemide [48], which is the most commonly administered drug in the non-serious ADRs group **(**S2 Table**)**.

The frequency of drug-related deaths identified in this study is 1.1%, which is consistent with that reported in the literature (0.1% to 13%) [32]; however, it is important to consider that there is wide variability in published values ​​and that, as previously mentioned, ADR underreporting at HIMFG is 93.9% [38] and, thus, the problem might be underestimated. Antineoplastic and immunomodulating agents use and febrile neutropenia occurred more commonly in patients whose outcome was drug-related death, which has already been widely studied in patients on cancer treatment [49,50]. On the other hand, 0.7% of patients recovered with some sequel, which is consistent with previous reports in the literature [51]. Up to 70.3% of ADRs resulted in hospital admission, which can be attributed to the fact that the main diagnosis of the patients in this study is of oncological nature and, in these patients, febrile neutropenia and sepsis are a cause of admissions to the emergency department [10].

As for the main groups of drugs that caused serious ADRs, it was the Antineoplastic and immunomodulating agents (81.2%), Cardiovascular system (8.0%) and Nervous system (4.2%) groups, while those that caused non-serious ADRs were from the Cardiovascular system (34.9%), Antiinfectives for systemic use (21.8%) and Nervous system (16.5%) groups. This is in contrast with reports in the literature, since according to the Uppsala Monitoring Center [16], the ATC groups of drugs that are associated with a higher frequency of ADRs in children are the Antiinfectives for systemic use (33%), Nervous system (28%) and Dermatologicals (12%) groups. However, in pediatric hospitals, the classes of drugs associated with ADRs according to severity level include those that have been reported to be of “low severity” such as antibiotics (34%), narcotic analgesics (11%) and anticonvulsants (10%), while antineoplastic agents (21%), anticonvulsants (19%) and narcotic analgesics (14%) are considered to be of “high severity” [52]. Despite methodological differences between the studies, the therapeutic groups that were associated with ADRs showed some contrasts between countries with the same National Income Level Per Capita as Mexico such as Malaysia, Colombia, Cuba and Uruguay [53]. In hospitalized children of Colombia, the three main medication groups that were associated with ADRs are Antibiotics, Respiratory system and Systemic hormonal preparations [54], while in Uruguay, the main groups were Antiinfectives for systemic use, Antiepileptics and Analgesics [55]. In both publications, Antineoplastic and immunomodulating agents were not reported to be associated with ADRs. On the other hand, in Cuban hospitalized children, the three main medication groups associated with ADRs were Antimicrobials, Non-opioid analgesics and Antineoplastic [56]. In hospitalized children of Malaysia, the most common were the Antiinfectives for systemic use, Nervous system and Respiratory system groups, while Antineoplastic and immunomodulating agents were at twelfth place [57].

### Study limitations

The limitations of the study that should be considered when interpreting the results include:

- ADR underreporting
- Barriers to identify some ADRs due to patients’ own characteristics; for example, headache caused by caffeine administration in neonates
- The explanation of some ADRs signs and symptoms can also be attributed to the highly complex diseases of HIMFG patients
- The findings here encountered cannot be generalized to other Mexican pediatric hospitals

## Conclusion

The safety information of the drugs that are marketed in the area of pediatrics in the national market is limited, and the need to strengthen Pharmacovigilance programs in hospitals is therefore evident. This is the first study in Mexico that provides an estimate of the impact of ADRs in hospitalized pediatric patients, which is highly important because the epidemiology of drug usage in children is different between countries. On the other hand, it contributes to the understanding of prescription patterns in Mexican pediatric hospitals, which should help the development of interventions to minimize the impact of ADRs on children.

## Supporting information

S1 Fig Annual frequency of ADRs based on hospital discharges. Numbers on top of the bars are the absolute numbers of total ADRs for the specified years.
(TIF)

S1 Table Anatomical Therapeutic Chemical (ATC) classification of suspect drugs related to serious ADRs. (DOCX)

S2 Table Anatomical Therapeutic Chemical (ATC) classification of suspect drugs related to non-serious ADRs. (DOCX)

S3 Table Original data used for data analysis. (XLSX)

S1 File Letter of approval by the institutional review board. (PDF)

S2 File Original protocol that was reviewed by the institutional review board. (PDF)
