# Supplementary figures and images for: Descriptive study of adverse drug reactions in a tertiary care pediatric hospital in México from 2014 to 2017
Source: PLoS One. 2020 Mar 24;15(3):e0230576. doi: 10.1371/journal.pone.0230576 (PMC7092985; doi:10.1371/journal.pone.0230576)

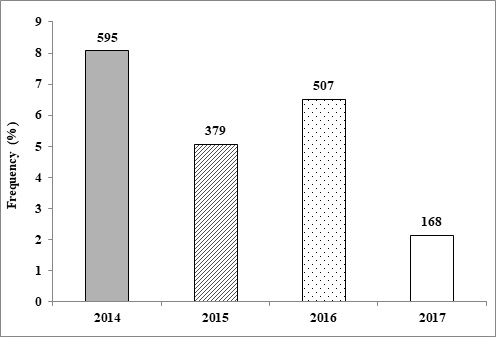

Supplement: S1 Fig — Numbers on top of the bars are the absolute numbers of total ADRs for the specified years. (TIF) [file pone.0230576.s001.tif]
