# Supplementary material for: Descriptive study of adverse drug reactions in a tertiary care pediatric hospital in México from 2014 to 2017
Source: PLoS One. 2020 Mar 24;15(3):e0230576. doi: 10.1371/journal.pone.0230576 (PMC7092985; doi:10.1371/journal.pone.0230576)
Supplement: S1 Table — (DOCX) [file pone.0230576.s002.docx]

| **S1 Table** Anatomical Therapeutic Chemical (ATC) classification of suspect drugs related to serious ADRs | | |
| --- | --- | --- |
| **ATC group** | **Drugs** | **Serious ADRs** |
| Antineoplastic and inmunomodulating agents (L)  1423 (81.2%) | Cytarabine 270 (19.0%), methotrexate 201 (14.1%), vincristine 199 (14.0%), cyclophosphamide 165 (11.6%), doxorubicin 88 (6.2%), daunorubicin 76 (5.3%), ifosfamide 76 (5.3%), etoposide 67 (4.7%), asparaginase 64 (4.5%), mercaptopurine 47 (3.3%), cisplatin 42 (3.0%), carboplatin 30 (2.1%), vinblastine 28 (2.0%), irinotecan 21 (1.5%), mitoxantrone 8 (0.6%), mifamurtide 7 (0.5%), dactinomycin 6 (0.4%), mycophenolic acid 6 (0.4%), tacrolimus 4 (0.3%), fluorouracil 3 (0.2%), imatinib 3 (0.2%), rituximab 2 (0.1%), antithymocyte immunoglobulin (rabbit) 2 (0.1%), tretinoin 2 (0.1%), azathioprine 1 (0.1%), cyclosporine 1 (0.1%), fludarabine 1 (0.1%), hydroxycarbamide 1 (0.1%), sorafenib 1(0.1%), topotecan 1 (0.1%). | Febrile neutropenia 775 (54.5%), sepsis 145 (10.2%), septic shock 100 (7.0%), pancytopenia 98 (6.9%), mucositis, unspecified 45 (3.2%), pancreatitis 22 (1.5%), thrombocytopenia 21 (1.5%), pneumonia 18 (1.3%), neutropenic colitis 16 (1.1%), diarrhea 15 (1.1%), abdomen – pain 10 (0.7%), anemia 8 (0.6%), infection 7 (0.5%), fever 6 (0.4%), tachycardia 6 (0.4%), vomiting 6 (0.4%), cellulitis 5 (0.4%), abnormal electrolytes 5 (0.4%), viral infection 5 (0.4%), seizures 4 (0.3%), epistaxis 4 (0.3%), cutaneous eruption 4 (0.3%), increased drug levels 4 (0.3%), hematuria 3 (0.2%), hemorrhage, unspecified 3 (0.2%), hypotension 3 (0.2%), ileus 3 (0.2%), renal insufficiency (acute) 3 (0.2%), neuropathy 3 (0.2%), allergic reaction 3 (0.2%), breathing – difficulty 3 (0.2%), hypovolemic shock 3 (0.2%), Stevens Johnson syndrome 3 (0.2%), adynamia 2 (0.1%), angioedema 2 (0.1%), asthenia 2 (0.1%), bradycardia 2 (0.1%), oral candidiasis 2 (0.1%), dehydration 2 (0.1%), edema 2 (0.1%), chills 2 (0.1%), extremities – pain 2 (0.1%), acute kidney failure 2 (0.1%), hypertension 2 (0.1%), proctitis 2 (0.1%), cough 2 (0.1%), renal tubular– disorder 2 (0.1%), gluteal abscess 1 (0.1%), perianal abscess 1 (0.1%), increased alanine aminotransferase 1 (0.1%), increased aspartate aminotransferase 1 (0.1%), mouth – ulceration 1 (0.1%), headache 1 (0.1%), hemorrhagic cystitis 1 (0.1%), cholangitis 1 (0.1%), colitis 1 (0.1%), increased blood creatinine 1 (0.1%), increased drug effect 1 (0.1%), effect – lack of 1 (0.1%), increased liver enzymes 1 (0.1%), palmar-plantar erythrodysesthesia 1 (0.1%), cardiorespiratory failure 1 (0.1%), multiple organ failure 1 (0.1%), pharyngitis 1 (0.1%), hemiparesis 1 (0.1%), hepatotoxic, effect 1 (0.1%), herpes zoster 1 (0.1%), neurological infection 1 (0.1%), upper respiratory infection 1 (0.1%), chronic renal insufficiency 1 (0.1%), respiratory insufficiency 1 (0.1%), irritability 1 (0.1%), leukopenia 1 (0.1%), lumbar – pain 1 (0.1%), tubular nephropathy 1 (0.1%), swollen eye 1 (0.1%), peritonitis 1 (0.1%), skin - lesion 1 (0.1%), increased transaminase, unspecified 1 (0.1%), thrombocythemia 1 (0.1%), anal ulcer 1 (0.1%), chicken pox 1 (0.1%), injection site – swelling 1 (0.1%). |
| Cardiovascular system (C)  141 (8.0%) | Furosemide 97 (68.8%), epinephrine 10 (7.1%), alprostadil 8 (5.7%), milrinone 6 (4.3%), propranolol 4 (2.8%), amiodarone 3 (2.1%), norepinephrine 3 (2.1%), captopril 2 (1.4%), spironolactone 2 (1.4%), verapamil 2 (1.4%), digoxin 1 (0.7%), hydralazine 1 (0.7%), levosimendan 1 (0.7%), losartan 1 (0.7%). | Abnormal electrolytes 89 (63.1%), apnea 7 (5%), hyperglycemia 7 (5%), metabolic alkalosis 6 (4.3%), hypotension 6 (4.3%), tachycardia 4 (2.8%), ventricular arrhythmia 2 (1.4%), acute renal insufficiency 2 (1.4%), pancreatitis 2 (1.4%), metabolic acidosis 1 (0.7%), respiratory alkalosis 1 (0.7%), anuria 1 (0.7%), cardiac arrhythmia 1 (0.7%), atelectasis 1 (0.7%), AV block 1 (0.7%), bradycardia 1 (0.7%), neonatal convulsions 1 (0.7%), respiratory depression 1 (0.7%), effect - lack of 1 (0.7%), fever 1 (0.7%), hypertension 1 (0.7%), hypothyroidism 1 (0.7%), myoclonus 1 (0.7%), nephrocalcinosis 1 (0.7%), thrombocytopenia 1 (0.7%). |
| Nervous system (N)  74 (4.2%) | Morphine 29 (39.2%), fentanyl 12 (16.2%), midazolam 11 (14.9%), paracetamol 4 (5.4%), diphenylhydantoin 4 (5.4%), risperidone 3 (4.1%), levetiracetam 2 (2.7%), tramadol 2 (2.7%), caffeine 1 (1.4%), dexmedetomidine hydrochloride 1 (1.4%), phenytoin 1 (1.4%), ketamine 1 (1.4%), metamizole sodium 1 (1.4%), propofol 1 (1.4%), thiopental 1 (1.4%). | Abnormal electrolytes 19 (25.7%), hypotension 8 (10.8%), bradycardia 5 (6.8%), respiratory depression 4 (5.4%), hypertension 3 (4.1%), tachycardia 3 (4.1%), metabolic acidosis 2 (2.7%), increased alanine aminotransferase 2 (2.7%), apnea 2 (2.7%), oliguria 2 (2.7%), drug withdrawal syndrome 2 (2.7%), somnolence 2 (2.7%), vomiting 2 (2.7%), abdomen - pain 1 (1.4%), decreased appetite 1 (1.4%), increased aspartate aminotransferase 1 (1.4%), bronchospasm 1 (1.4%), seizures 1 (1.4%), effect – lack of 1 (1.4%), hemorrhage, unspecified 1 (1.4%), hyperglycemia 1 (1.4%), hypoventilation 1 (1.4%), antidiuretic hormone – disorder 1 (1.4%), leukopenia 1 (1.4%), increased lipase 1 (1.4%), dizziness 1 (1.4%), oxygen - decreased saturation 1 (1.4%), decreased therapeutic response 1 (1.4%), sialorrhea 1 (1.4%), DRESS syndrome 1 (1.4%), tachypnea 1 (1.4%). |
| Antiinfectives for systemic use (J)  39 (2.2%) | Amphotericin B 12 (30.8%), cefepime 7 (17.9%), cefalotin 3 (7.7%), metronidazole 3 (7.7%), caspofungin 2 (5.1%), ceftriaxone 2 (5.1%), clindamycin 2 (5.1%), piperacillin/tazobactam 2 (5.1%), immunoglobulins normal human 1 (2.6%), ganciclovir 1 (2.6%), meropenem 1 (2.6%), rifampicin/isoniazid/pyrazinamide/ethambutol 1 (2.6%), valganciclovir 1(2.6%), vancomycin 1 (2.6%). | Abnormal electrolytes 11 (28.2%), cutaneous eruption 4 (10.3%), increased alanine aminotransferase 3 (7.7%), pancreatitis 3 (7.7%), tachycardia 3 (7.7%), hypertension 2 (5.1%), metabolic acidosis 1 (2.6%), anaphylaxis 1 (2.6%), cardiac arrhythmia SE 1(2.6%), increased liver enzymes 1 (2.6%), decreased plasma fibrinogen 1 (2.6%), fever 1 (2.6%), increased gamma-glutamyl transferase 1 (2.6%), hemorrhage, unspecified 1 (2.6%), hyperbilirubinemia 1 (2.6%), hypotension 1 (2.6%), breathing – difficulty 1 (2.6%), shock 1 (2.6%), increased coagulation time 1 (2.6%). |
| Systemic hormonal preparations, excl. sex hormones and insulins (H)  39 (2.2%) | Dexamethasone 22 (56.4%), methylprednisolone 11 (28.2%), hydrocortisone 3 (7.7%), prednisone 2 (5.1%), thiamazole 1 (2.6%). | Abnormal electrolytes 8 (20.5%), hypertension 5 (12.8%), febrile neutropenia 5 (12.8%), respiratory alkalosis 4 (10.3%), hyperglycemia 3 (7.7%), increased alanine aminotransferase 2 (5.1%), sepsis 2 (5.1%), decreased amylase 1 (2.6%), cardiac arrhythmia, unspecified 1 (2.6%), diabetic ketoacidosis 1 (2.6%), fever 1 (2.6%), GI hemorrhage 1 (2.6%), intracranial hypertension 1 (2.6%), increased lipase 1 (2.6%), pneumonia 1 (2.6%), septic shock 1 (2.6%), tachycardia 1 (2.6%). |
| Various (V)  15 (0.9%) | Iopromide 15 (100%). | Headache 3 (20%), chills 3 (20%), fever 3 (20%), vertigo 3 (20%), vomiting 3 (20%). |
| Blood and blood forming organs (B)  10 (0.6%) | Heparin 4 (40.0%), enoxaparin 2 (20.0%), acenocoumarol 1 (10.0%), aminocaproic acid 1 (10.0%), arginine 1 (10.0%), erythropoietin 1 (10.0%). | Pulmonary hemorrhage 2 (20%), hemorrhage, unspecified 2 (20%), abnormal electrolytes 1 (10%), epistaxis 1 (10%), GI hemorrhage 1 (10%), retinopathy 1 (10%), thrombocytopenia 1 (10%), thrombosis 1 (10%). |
| Musculo-skeletal system (M)  7 (0.4%) | Vecuronium 4 (57.1%), rocuronium bromide 2 (28.6%), cisatracurium 1 (14.3%). | Hypotension 2 (28.6%), bronchospasm 1 (14.3%), cutaneous eruption 1 (14.3%), muscle relaxation 1 (14.3%), tachycardia 1 (14.3%), tachypnea 1 (14.3%). |
| Alimentary tract and metabolism (A)  4 (0.2%) | Omeprazole 2 (50.0%), cisapride 1 (25.0%), lactulose 1 (25.0%). | Diabetes insipidus 1 (25%), diarrhea 1 (25%), abnormal electrolytes 1 (25%), extrasystoles 1 (25%). |
| Respiratory system (R)  1 (0.1%) | Natural phospholipids 1 (100%). | Hypotension 1 (100%). |
