# Supplementary material for: Descriptive study of adverse drug reactions in a tertiary care pediatric hospital in México from 2014 to 2017
Source: PLoS One. 2020 Mar 24;15(3):e0230576. doi: 10.1371/journal.pone.0230576 (PMC7092985; doi:10.1371/journal.pone.0230576)
Supplement: S2 Table — (DOCX) [file pone.0230576.s003.docx]

| **S2 Table** Anatomical Therapeutic Chemical (ATC) classification of suspect drugs related to non-serious ADRs | | |
| --- | --- | --- |
| **ATC group** | **Drugs** | **Non-serious ADRs** |
| Cardiovascular system (C)  144 (34.9%) | Furosemide 92 (63.9%), epinephrine 19 (13.2%), milrinone 6 (4.2%), alprostadil 5 (3.5%), spironolactone 4 (2.8%), norepinephrine 4 (2.8%), amiodarone 3 (2.1%), dobutamine 3 (2.1%), propranolol 2 (1.4%), atorvastatin 1 (0.7%), bumetanide 1 (0.7%), captopril 1 (0.7%), digoxin 1 (0.7%), hydralazine 1 (0.7%), losartan 1 (0.7%). | Abnormal electrolytes 86 (59.7%), hyperglycemia 18 (12.5%), fever 5 (3.5%), increased aspartate aminotransferase 4 (2.8%), tachycardia 4 (2.8%), increased alanine aminotransferase 3 (2.1%), hypotension 3 (2.1%), metabolic alkalosis 2 (1.4%), abnormal blood glucose 2 (1.4%), thrombocytopenia 2 (1.4%), vomiting 2 (1.4%), respiratory alkalosis 1 (0.7%), decreased appetite 1 (0.7%), bradycardia 1 (0.7%), sinus bradycardia 1 (0.7%), headache 1 (0.7%), exaggerated diuresis 1 (0.7%), cutaneous eruption 1 (0.7%), hypertension 1 (0.7%), hypoalbuminemia 1 (0.7%), oliguria 1 (0.7%), decreased urine flow 1 (0.7%), increased urine volume 1 (0.7%), somnolence 1 (0.7%). |
| Antiinfectives for systemic use (J)  90(21.8%) | Amphotericin B 14 (15.6%), meropenem 11 (12.2%), clindamycin 10 (11.1%), cefepime 8 (8.9%), vancomycin 8 (8.9%), cefotaxime 6 (6.7%), ceftriaxone 6 (6.7%), cefalotin 5 (5.6%), ampicillin 4 (4.4%), clarithromycin 3 (3.3%), efavirenz/emtricitabine/tenofovir 2 (2.2%), metronidazole 2 (2.2%), rifampicin 2 (2.2%), rifampicin/ isoniazid/pyrazinamide/ethambutol 2 (2.2%), trimethoprim/sulfamethoxazole 2 (2.2%), amikacin 1 (1.1%), amoxicillin 1 (1.1%), amoxicillin/clavulanic acid 1 (1.1%), doxycycline 1 (1.1%), piperacillin/tazobactam 1(1.1%). | Cutaneous eruption 20 (22.2%), increased alanine aminotransferase 5 (5.6%), increased aspartate aminotransferase 5 (5.6%), vomiting 5 (5.6%), tachycardia 4 (4.4%), anemia 3 (3.3%), apnea 3 (3.3%), eosinophilia 3 (3.3%), fever 3 (3.3%), nausea 3 (3.3%), thrombocytopenia 3 (3.3%), abnormal electrolytes 2 (2.2%), decreased hemoglobin 2 (2.2%), hyperbilirubinemia 2 (2.2%), abdomen, pain 1 (1.1%), abdominal discomfort 1 (1.1%), metabolic acidosis 1 (1.1%), bandemia 1 (1.1%), bradycardia 1 (1.1%), headache 1 (1.1%), diarrhea 1 (1.1%), edema 1 (1.1%), edema of the neck 1 (1.1%), enterocolitis 1 (1.1%), increased liver enzymes 1 (1.1%), chills 1 (1.1%), increased gamma-glutamyl transferase 1 (1.1%), gastritis 1 (1.1%), abnormal blood glucose 1 (1.1%), hyperglycemia 1 (1.1%), hypertension 1 (1.1%), hypotension 1 (1.1%), irritability 1 (1.1%), increased plasma lactate 1 (1.1%), leukocytosis 1 (1.1%), leukopenia 1 (1.1%), respiratory obstruction, unspecified 1 (1.1%), urine, color change 1 (1.1%), red man syndrome 1 (1.1%), increased prothrombin time 1 (1.1%), increased triglycerides 1 (1.1%). |
| Nervous system (N)  68 (16.5%) | Morphine 18 (26.5%), paracetamol 9 (13.2%), fentanyl 9 (13.2%), atomoxetine 6 (8.8%), midazolam 5 (7.4%), caffeine 4 (5.9%), tramadol 4 (5.9%), dexmedetomidine hydrochloride 3 (4.4%), ergotamine/caffeine 3 (4.4%), levetiracetam 2 (2.9%), thiopental 2 (2.9%), ketamine 1 (1.5%), methylphenidate 1 (1.5%), propofol 1 (1.5%). | Abnormal electrolytes 11 (16.2%), tachycardia 7 (10.3%), hypotension 6 (8.8%), cutaneous eruption 5 (7.4%), somnolence 4 (5.9%), vomiting 4 (5.9%), increased aspartate aminotransferase 2 (2.9%), bradycardia 2 (2.9%), respiratory depression 2 (2.9%), constipation 2 (2.9%), leukocytosis 2 (2.9%), nausea 2 (2.9%), abdomen, pain 1 (1.5%), metabolic acidosis 1 (1.5%), aggressiveness 1 (1.5%), increased alanine aminotransferase 1 (1.5%), anemia 1 (1.5%), anxiety 1 (1.5%), decreased appetite 1 (1.5%), apnea 1 (1.5%), asthenia 1 (1.5%), mood swings 1 (1.5%), headache 1 (1.5%), costiveness 1 (1.5%), diabetes insipidus 1 (1.5%), diaphoresis 1 (1.5%), irritability 1 (1.5%), emotional lability 1 (1.5%), leukopenia 1 (1.5%), dizziness 1 (1.5%), oliguria 1 (1.5%). |
| Systemic hormonal preparations, excl. sex hormones and insulins (H)  35 (8.5%) | Methylprednisolone 26 (74.3%), dexamethasone 6 (17.1%), prednisone 2 (5.7%), vasopressin 1 (2.9%). | Hyperglycemia 11 (31.4%), abnormal electrolytes 5 (14.3%), leukocytosis 4 (11.4%), increased alanine aminotransferase 3 (8.6%), increased aspartate aminotransferase 3 (8.6%), increased urine calcium 2 (5.7%), abnormal blood glucose 2 (5.7%), diabetes mellitus 1 (2.9%), fever 1 (2.9%), decreased hemoglobin 1 (2.9%), hypertension 1 (2.9%), neutrophilia 1 (2.9%). |
| Antineoplastic and inmunomodulating agents (L)  21 (5.1%) | Mycophenolic acid 4 (19.0%), filgrastim 3 (14.3%), methotrexate 3 (14.3%), tacrolimus 2 (9.5%), vincristine 2 (9.5%), fluorouracil 1 (4.8%), cyclosporine 1 (4.8%), cisplatin 1 (4.8%), ifosfamide 1 (4.8%), picibanil 1 (4.8%), sirolimus 1(4.8%), anti-thymocyte immunoglobulin (rabbit) 1 (4.8%). | Viral infection 3 (14.3%), leukopenia 2 (9.5%), mucositis, unspecified 2 (9.5%), febrile neutropenia 1 (4.8%), bandemia 1(4.8%), increased blood creatinine 1(4.8%), abnormal electrolytes 1(4.8%), epistaxis 1(4.8%), fever 1(4.8%), paralytic ileus 1(4.8%), leukocytosis 1(4.8%), nausea 1(4.8%), neuropathy 1(4.8%), oliguria 1(4.8%), increased triglycerides 1(4.8%), thrombocytosis 1(4.8%), vomiting 1(4.8%). |
| Alimentary tract and metabolism (A)  20 (4.8%) | Metformin 11 (55.0%), cisapride 2 (10.0%), domperidone 2 (10.0%), calcium gluconate 2 (10.0%), omeprazole 2 (10.0%), citric acid 1 (5.0%). | Diaphoresis 2 (10%), abnormal electrolytes 2 (10%), nausea 2 (10%), abdomen, pain 1 (5%), apnea 1 (5%), headache 1 (5%), fainting 1 (5%), chills 1 (5%), spasms 1 (5%), constipation 1 (5%), extravasation 1 (5%), leukocytosis 1 (5%), dizziness 1 (5%), paleness 1 (5%), tachycardia 1 (5%), tremor of extremities 1 (5%), tetany 1 (5%). |
| Musculo-skeletal system (M)  12 (2.9%) | Ibuprofen 4 (33.3%), allopurinol 3 (25.0%), naproxen 2 (16.7%), vecuronium 2 (16.7%), tizanidine 1 (8.3%). | Cutaneous eruption 2 (16.7%), tachycardia 2 (16.7%), anemia 1 (8.3%), bradycardia 1 (8.3%), epigastric, food-unrelated pain 1 (8.3%), esophageal hemorrhage 1 (8.3%), hyperuricemia 1 (8.3%), leukopenia 1 (8.3%), nausea 1 (8.3%), neutropenia 1 (8.3%). |
| Blood and blood forming organs (B)  7 (1.7%) | Enoxaparin 3 (42.9%), potassium chloride 1 (14.3%), heparin 1 (14.3%), magnesium sulfate 1 (14.3%), vitamin K 1 (14.3%). | Cutaneous eruption 2 (28.6%), hemorrhage, unspecified 2 (28.6%), abnormal electrolytes 1 (14.3%), somnolence 1 (14.3%), increased prothrombin time (14.3%). |
| Genito urinary system and sex hormones (G)  7 (1.7%) | Levonorgestrel/ethinyl estradiol 3 (42.9%), drospirenone/ethinyl estradiol 2 (28.6%), cyproterone/ethinyl estradiol 1 (14.3%), sildenafil 1 (14.3%). | Acne 2 (28.6%), insomnia 1 (14.3%), irritability 1 (14.3%), intermenstrual spotting 1 (14.3%), menstruation, exaggerate flow 1 (14.3%), tachycardia 1 (14.3%). |
| Antiparasitic products, insecticides and repellents (P)  4 (1.0%) | Hydroxychloroquine 4 (100%). | Headache 1 (25%), retro-orbital pain 1 (25%), insomnia 1 (25%), dizziness 1 (25%). |
| Respiratory system (R)  3 (0.7%) | Aminophylline 2 (66.7%), chlorphenamine 1 (33.3%) | Tachycardia 2 (66.7%), cutaneous eruption 1 (33.3%). |
| Sensory organs (S)  1 (0.2%) | Atropine 1 (100%) | Tachycardia 1 (100%). |
| Various (V)  1 (0.2%) | Contrast medium 1 (100%) | Nausea 1 (100%). |
