# Supplementary material for: Descriptive study of adverse drug reactions in a tertiary care pediatric hospital in México from 2014 to 2017
Source: PLoS One. 2020 Mar 24;15(3):e0230576. doi: 10.1371/journal.pone.0230576 (PMC7092985; doi:10.1371/journal.pone.0230576)
Supplement: S1 File — (PDF) [file pone.0230576.s005.pdf]

SALUD
SECRETARÍA DE SALUD

HOSPITAL INFANTIL DE MÉXICO FEDERICO GÓMEZ
INSTITUTO NACIONAL DE SALUD

DIRECCIÓN GENERAL

75 años
¡Haciéndolo mejor!

Ciudad de México, a 23 de marzo de 2018 DG/1000/ **197** /2018

Dra. Olga Magdala Morales Ríos
Departamento de Investigación Clínica
Presente

Informo a usted, que los Comités de Investigación, Ética y Bioseguridad, después de haber revisado su protocolo **HIM 2018-003** "Estudio Descriptivo de las notificaciones de reacción adversa a medicamentos del Programa Electrónico de Farmacovigilancia del Hospital Infantil de México Federico Gómez", han emitido el dictamen de:

APROBADO

En los términos y condiciones señalados por dichos Comités. Por lo anterior, se autoriza su desarrollo.

Atentamente

Dr. José Alberto García Aranda
Director General

Con copia:
Lic. Martha Reynoso Robles. Jefa del Departamento Administrativo de Control y Gestión a la Investigación.

JAGA/JGE/JGO/ash

CERTIFICACIÓN SiNaCEAM

INSTITUTO NACIONAL DE SALUD AFILIADO A LA UNAM

DR. MÁRQUEZ 162, COL. DOCTORES. DEL. CUAUHTÉMOC, C.P. 06720 MÉXICO D.F.
CONMUTADOR: 5228-9917 EXT. 4314 Y 4100
www.himfg.edu.mx
