# Supplementary material for: Descriptive study of adverse drug reactions in a tertiary care pediatric hospital in México from 2014 to 2017
Source: PLoS One. 2020 Mar 24;15(3):e0230576. doi: 10.1371/journal.pone.0230576 (PMC7092985; doi:10.1371/journal.pone.0230576)
Supplement: S2 File — (PDF) [file pone.0230576.s006.pdf]

HOSPITAL INFANTIL DE MÉXICO FEDERICO GÓMEZ

Departamento de Evaluación y Análisis de Medicamentos

Título del trabajo:

Estudio Descriptivo de las notificaciones de reacción adversa a medicamentos del Programa Electrónico de Farmacovigilancia del Hospital Infantil de México Federico Gómez

Investigador Responsable
Dra. Olga Magdala Morales Ríos

Investigador Suplente
Dr. Luis Jasso Gutiérrez

Investigadores Participantes

pQFB. Carlos Andrés García Ruíz
Dr. Victor Olivar López
Dr. Carlo Egysto Cicero Oneto
Dra. Maribelle Hernández Hernández
Dra. Dina Villanueva García
Dr. Rodolfo Jiménez Juárez

Versión 2.0
Ciudad de México, Abril 2018

I. ÍNDICE

I. ÍNDICE 2
II. RESUMEN 3
III. ANTECEDENTES 4
V. PLANTEAMIENTO DEL PROBLEMA Y JUSTIFICACIÓN 10
VI. PREGUNTAS DE INVESTIGACIÓN 10
VII. OBJETIVOS 10
VIII. METODOLOGÍA Y PLAN DE ANÁLISIS DE LOS DATOS 11
IX. LIMITACIONES DEL ESTUDIO 19
X. BIBLIOGRAFÍA 19
XI. ANEXOS 21

II. RESUMEN

Antecedentes: Las actividades de Farmacovigilancia en pediatría son muy importantes porque los niños pueden ser más vulnerables que los adultos a presentar Reacciones Adversas a Medicamentos (RAMs) debido: **a)** a la prescripción de medicamentos unlicensed y off label, **b)** a la exposición a fármacos durante la etapa prenatal y la lactancia materna, **c)** a que los niños pueden reaccionar diferente a los adultos a la administración de los medicamentos, y **d)** a que a los pacientes pediátricos hospitalizados se les pueden administrar más de 25 medicamentos durante su estancia. En la población pediátrica las RAMs tienen impacto en la morbilidad, la mortalidad y en los costos, y su frecuencia oscila desde el 0.14% al 21.5%, pero específicamente en las Unidades de Cuidados Intensivos los porcentajes van desde el 7% al 36.6% y en Urgencias es del 26.4%. Por otro lado, las actividades de Farmacovigilancia del Hospital Infantil de México Federico Gómez (HIMFG) están coordinadas por el Centro Institucional de Farmacovigilancia, que entre otras cosas, es el encargado de enviar electrónicamente las notificaciones de RAMs al Centro Nacional de Farmacovigilancia. Sin embargo, en México no hay datos en población pediátrica sobre las características de las RAMs así como de los medicamentos sospechosos más frecuentemente involucrados.

Objetivos

1.- Describir las manifestaciones clínicas de las RAMs que los médicos del HIMFG notificaron en el Programa Electrónico de Farmacovigilancia

2.- Describir los medicamentos sospechosos involucrados en las RAMs que los médicos del HIMFG notificaron en el Programa Electrónico de Farmacovigilancia

3.- Describir las características de las RAMs (consecuencia de la RAM, calidad de la información, causalidad, severidad y gravedad) de acuerdo a la NOM-220-SSA1-2012 de Farmacovigilancia que los médicos del HIMFG notificaron en el Programa Electrónico de Farmacovigilancia

Metodología y plan de análisis de los datos

Diseño: Análisis secundario de la base de datos del Programa Electrónico de Farmacovigilancia del HIMFG

Población de estudio: 1,500 notificaciones de RAMs

Plan de análisis estadístico:

Análisis descriptivo: número de casos (%), media (IC 95%) y mediana (percentiles 25-75)

III. ANTECEDENTES

Los orígenes de la Farmacovigilancia son de los años 1959 a 1961, cuando pediatras de Alemania, Inglaterra y Estados Unidos de Norteamérica atendieron a niños con deformaciones en sus extremidades que se parecían a las de las focas (focomelia). Derivado del análisis de los casos, se encontró que la epidemia obedeció a la administración de talidomida, que era un fármaco indicado como hipnótico a mujeres en etapas tempranas de la gestación **[1]**. Ante esta situación, en 1962 la Organización Mundial de la Salud (OMS) elaboró un programa para la promoción de la seguridad de los medicamentos y en 1964 solicitó a los países participantes que establecieran sus propios sistemas de registro sobre los efectos indeseables de los medicamentos, además consideró establecer un centro internacional con su respectivo programa encargado de monitorear estos efectos indeseables a través de una comunicación sistemática con los centros nacionales. Después de varias asambleas, finalmente se inició en 1968 el proyecto piloto de investigación para el monitoreo internacional de los medicamentos y su financiamiento corrió a cargo del gobierno de Estados Unidos de Norteamérica. Hacia la mitad de la década de los setenta del siglo pasado, surgió la amenaza de descontinuar el programa debido a problemas financieros y cambio de prioridades en la OMS, pero en 1978 el gobierno sueco se hizo cargo del financiamiento y el centro migró a Uppsala; con 24 países participantes. Posteriormente, en una reunión entre la OMS y el Gobierno de Suecia, se acordó que la OMS sería la responsable de las cuestiones políticas del programa, mientras que la responsabilidad operativa recaería en el Centro de Monitoreo de Uppsala (CMU) **[1]**. Hasta enero del 2018 son 127 países los miembros oficiales (entre los que se encuentra México) y 29 países los miembros asociados **[2]**. El CMU define a la Farmacovigilancia como “la ciencia y actividades relacionadas a la detección, medición, entendimiento y prevención de efectos adversos o cualquier otro problema relacionado a fármacos” y a las Reacciones Adversas a Medicamentos (RAMs) como “un efecto nocivo sospechoso de ser causado por un medicamento” **[3]**. Mientras que la NOM-220-SSA1-2012 “Instalación y operación de la farmacovigilancia” define a una RAM como “cualquier reacción nociva no intencionada que aparece a dosis normalmente empleadas en el ser humano para la profilaxis, el diagnóstico o el tratamiento, o para la modificación de una función fisiológica” **[4]**.

En Pediatría la Farmacovigilancia es muy importante debido a que los niños comparados con los adultos pueden tener una mayor vulnerabilidad en presentar RAMs debido a: **a)** la prescripción de medicamentos unlicensed (medicamentos que no están autorizados por las Agencias Reguladoras para su uso en humanos, como es el caso de los medicamentos

preparados por la farmacia del hospital o por cualquier otra farmacia) y off label (medicamentos cuyos usos no están detallados en el resumen de características del producto farmacéutico como indicación terapéutica, dosis, forma farmacéutica) [5]; **b)** la exposición a fármacos durante la etapa prenatal y la lactancia materna [6], **c)** que los niños pueden reaccionar diferente a los adultos a la administración de los medicamentos, lo cual se puede explicar por los cambios en la absorción, distribución, metabolismo y excreción [7] y **d)** que a los pacientes pediátricos hospitalizados se les pueden administrar más de 25 medicamentos durante su estancia [8].

En los niños hospitalizados la frecuencia de RAMs oscila desde el 0.14% al 21.5% [9-10], pero específicamente en las Unidades de Cuidados Intensivos los porcentajes van desde el 7% al 36.6% [11-13] y en Urgencias es del 26.4% [14]. Mientras que de acuerdo a lo reportado por el CMU, el 7.7% de las notificaciones que reciben son de la población pediátrica [15]. Como se puede ver en la **figura 1**, las frecuencias de RAMs reportadas en pediatría tienen una amplia variabilidad, misma que se puede explicar por las diferentes metodologías empleadas para llevar a cabo la Farmacovigilancia, por las áreas en las que se llevó a cabo la investigación, y la duración del estudio, entre otras variables. En México no se encontraron datos de frecuencia de RAMs en los niños.

Fig 1. Frecuencia de RAMs en los niños Hospitalizados, en Unidades de Cuidados Intensivos, Urgencias y el Centro de Monitoreo de Uppsala.

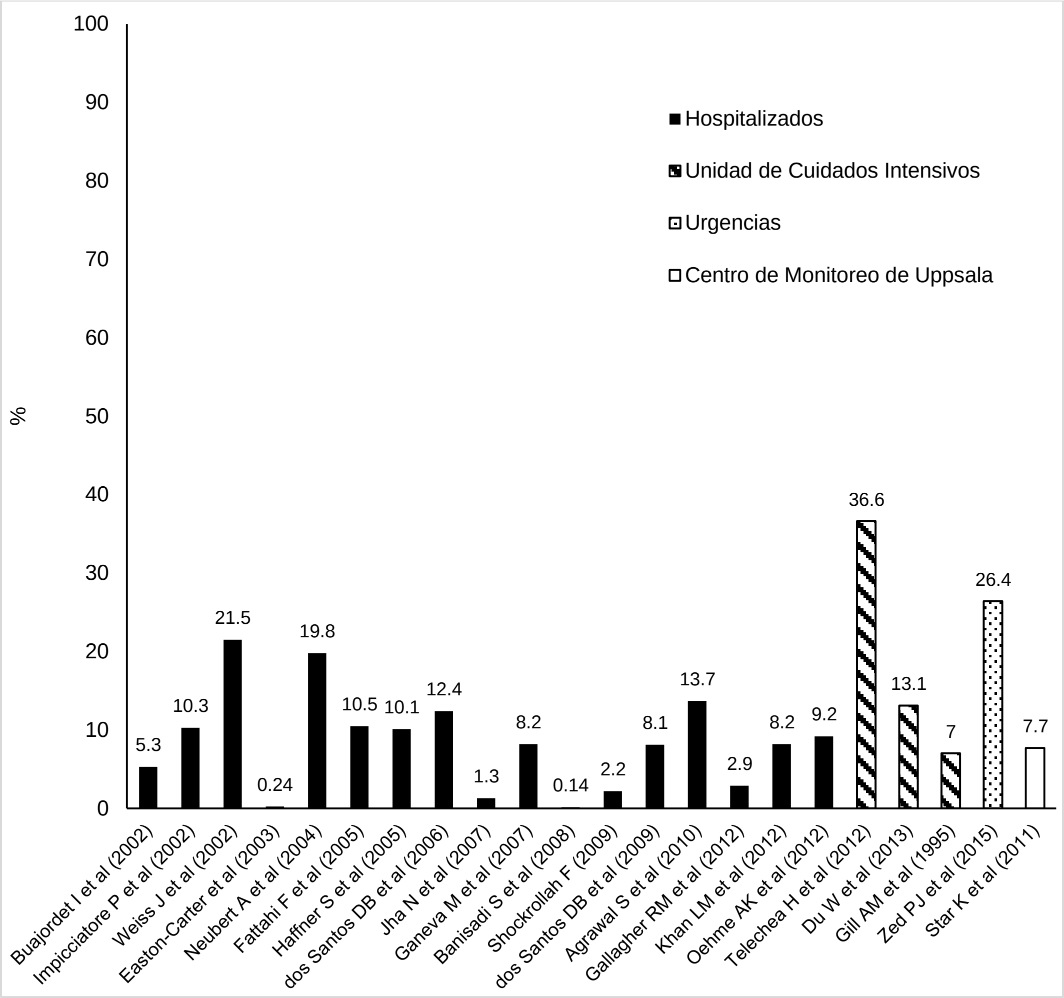

Fuente: Modificado de Khan L. Comparative epidemiology of hospital-acquired adverse drug reactions in adults and children and their impact on cost and hospital stay--a systematic review. Eur J Clin Pharmacol. 2013;69:1985-96

Las RAMs generan un gran impacto en la morbilidad, la mortalidad [16-17] y en los costos [18]. Un estudio que incluyó pacientes pediátricos admitidos entre los años 2000 y 2007 al Hospital for Sick Children y Children's Hospital Boston con diagnóstico de Síndrome de Stevens-Johnson y Necrolisis Epidérmica Tóxica, menciona que el 45% de estos niños presentaron secuelas a largo plazo y el 2% falleció, además que los fármacos más frecuentemente involucrados fueron los anticonvulsivantes (29%) seguidos por los agentes antimicrobianos (20%) y los quimioterapéuticos (2%) [16-17]. De acuerdo al Centro de Monitoreo de Uppsala [15] los grupos de medicamentos a los que se asocia una mayor

frecuencia de RAMs son Anti infecciosos de uso sistémico (33%), del Sistema Nervioso (28%) y Dermatológicos (12%). Además, en base a la clasificación por órganos y sistemas, las manifestaciones más reportadas para niños fueron de Trastornos de la piel y subcutáneo (35%), seguido de Trastornos generales y Alteraciones en el lugar de administración (20%), y de Trastornos del Sistema Nervioso (19%).

Con lo relacionado a los costos hospitalarios, como es de esperarse una RAM severa tiene un mayor costo que una RAM moderada [18], lo cual se puede observar en la **tabla 1**.

Tabla 1. Costos para el hospital por caso de Reacción Adversa a Medicamentos

	**RAM Moderada (Dólares)**	**RAM Severa (Dólares)**
Costos de hospitalización	17.62	26.61
Costos de honorarios de los profesionales de la salud	36.51	112.14
Costos de los medicamentos	28.81	311.86
Costos de los diagnósticos	27.96	91.10
Otros[a]	5.08	15.25
Costos de las visitas de seguimiento	13.80	52.28
**Costo total por caso de RAM**	**129.81**	**609.27**

a: Promedio de costos de electricidad, ropa, agua, depreciación del equipo y edificio, gastos de mantenimiento

Fuente: Modificado de Oshikoya KA, Chukwura H, Njokanma OF, Senbanjo IO, Ojo I. Incidence and cost estimate of treating pediatric adverse drug reactions in Lagos, Nigeria. Sao Paulo Med J. 2011;129(3):153-64

Para llevar a cabo las actividades de Farmacovigilancia existen diferentes métodos entre los que se encuentra la Notificación Espontanea o también llamada Reporte Espontaneo, el cual se define como el método empleado en farmacovigilancia consistente en el reporte que hacen los profesionales de la salud, pacientes o sus familiares, en lo concerniente a las sospechas de RAMs [4]. La Notificación Espontánea, es el método utilizado por el CMU para la detección temprana de nuevas señales y para la detección de RAMs graves y de poca frecuencia [19].Tiene la ventaja de ser un método económico y simple de operar, cubre todos los fármacos que están en el mercado incluyendo toda la población de pacientes así como subgrupos especiales como es el caso de los niños [20]. A pesar de tener muchas ventajas, es importante reconocer que con la Notificación Espontanea se pueden generar falsas señales y no se puede obtener información sobre datos de frecuencia, lo que traería como consecuencia que la magnitud del problema se subestime. Otra limitante es el subreporte, que disminuye su efectividad retrasando la detección de nuevas RAMs [20] y que puede oscilar desde el 6 hasta

el 100% **[21]**. Específicamente, en el Departamento de Urgencias del Hospital Infantil de México Federico Gómez (HIMFG) el subreporte es del 93.9% **[22]**.

En México, el Centro Nacional de Farmacovigilancia (CNF) dependiente de la Comisión Federal para la Protección contra Riesgos Sanitarios (COFEPRIS) tiene la responsabilidad de dirigir y coordinar el Programa Permanente de Farmacovigilancia (PPF) en base a lo establecido por el CMU. En las actividades del PPF participan principalmente Centros de Farmacovigilancia en cada entidad federativa (CE), Centros Institucionales (CI), Unidades de Farmacovigilancia Hospitalaria (UFH) y la Industria Química Farmacéutica **[4]**. Hasta el año 2014 participaban 19 CI, entre ellos el del HIMFG que se registró a inicios del año 2011. Particularmente, las actividades de Farmacovigilancia del HIMFG están basadas en la Notificación Espontánea, por lo que en el año 2008 el Centro Institucional de Farmacovigilancia del HIMFG (CIHIMFG) diseñó un programa para el llenado en línea de los formatos de RAMs denominado SISFAR el cual tiene la ventaja de ser amigable y su captura consume menos tiempo comparado con el llenado manual **[23]**. A inicios del año 2009 el CIHIMFG instaló el SISFAR en las áreas de hospitalización de: Oncología, Terapia Intensiva, Neonatología, Nefrología, Medicina Interna, Infectología, Gastroenterología, Cardiología, Reumatología, Urgencias, Cirugía General y de Tórax, Especialidades y Cirugía Cardiovascular. Después que los médicos realizan su reporte de sospecha de RAM empleando el SISFAR éste se recibe en el CIHIMFG en donde un farmacéutico las ingresa al Sistema Electrónico de COFEPRIS llamado Sistema de Captura y Evaluación de las Notificaciones de Sospechas de Reacciones Adversas de los Medicamentos y ETAV´S (SISCE v1.2.2014) y analiza cada notificación de acuerdo a la NOM-220-SSA1-2012 **[4]** respecto a la causalidad, gravedad, severidad y calidad de la información. Una vez que el farmacéutico las analiza, las envía de forma electrónica al CNF (**Figura 2**).

Fig 2. Sistema Electrónico de Farmacovigilancia del Hospital Infantil de México Federico Gómez. 1) En cada área de hospitalización los médicos detectan las RAM y capturan las notificaciones en el Sistema de Farmacovigilancia (SISFAR). **2)** El Centro Institucional de Farmacovigilancia del HIMFG recibe las notificaciones en el programa SISFAR. Para analizar las notificaciones de RAMs de acuerdo a la NOM-220-SSA1-2012 el Centro Institucional de Farmacovigilancia utiliza el programa electrónico de COFEPRIS SISCE v1.2.2014. El envío a COFEPRIS se realiza electrónicamente. **3)** La COFEPRIS recibe las notificaciones de RAMs y las envía a Uppsala Monitoring Centre.

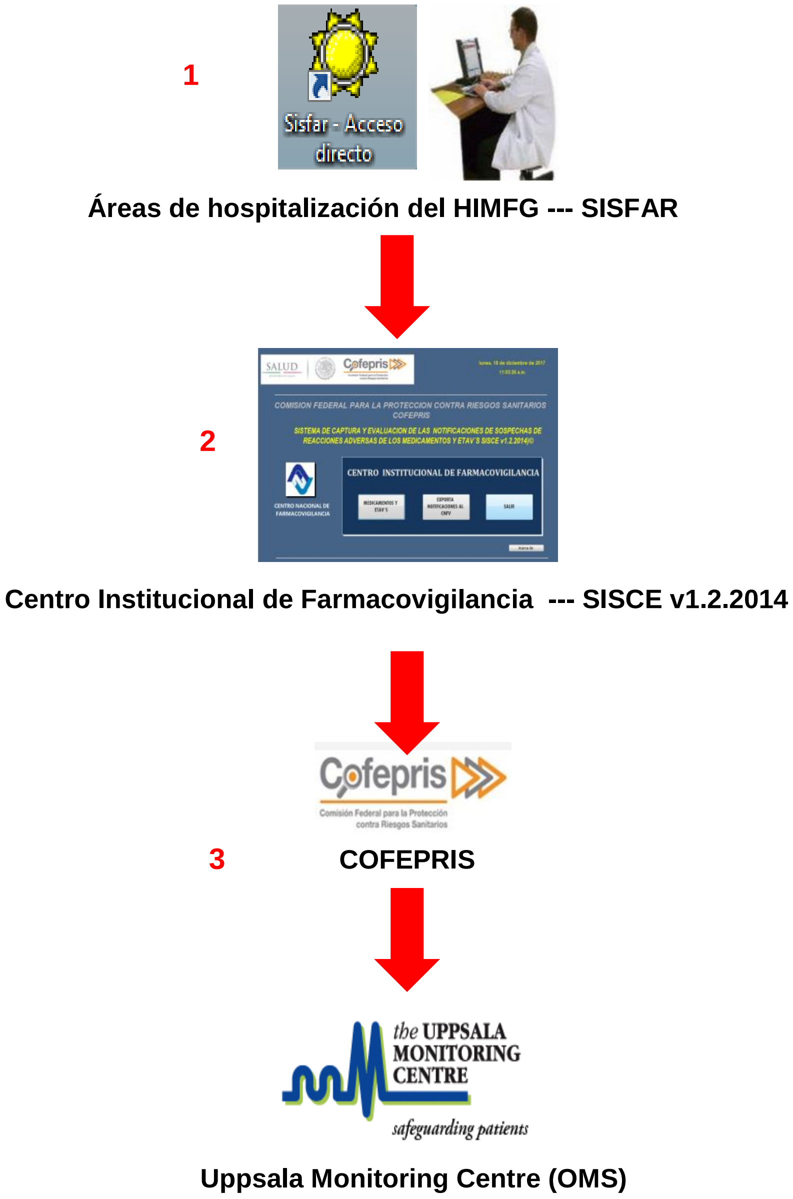

Fuente: Elaboración propia

V. PLANTEAMIENTO DEL PROBLEMA Y JUSTIFICACIÓN

Como se mencionó previamente, las actividades de Farmacovigilancia en pediatría son muy importantes porque los niños pueden ser más vulnerables que los adultos a presentar RAMs. En la población pediátrica las RAMs tienen impacto en la morbilidad, la mortalidad y en los costos y su frecuencia oscila desde el 0.14% al 21.5%, pero específicamente en las Unidades de Cuidados Intensivos los porcentajes van desde el 7% al 36.6% y en Urgencias es del 26.4%. En México no hay datos en población pediátrica sobre las manifestaciones clínicas que se presentan así como de los medicamentos sospechosos más frecuentemente involucrados.

VI. PREGUNTAS DE INVESTIGACIÓN

1.- ¿Cuáles son las manifestaciones clínicas de las RAMs que los médicos del HIMFG notificaron en el Programa Electrónico de Farmacovigilancia?

2.- ¿Cuáles son los medicamentos sospechosos involucrados en las RAMs que los médicos del HIMFG notificaron en el Programa Electrónico de Farmacovigilancia?

3.- ¿Cuáles son las características de las RAMs de acuerdo a la NOM-220-SSA1-2012 de Farmacovigilancia que los médicos del HIMFG notificaron en el Programa Electrónico de Farmacovigilancia?

VII. OBJETIVOS

1.- Describir las manifestaciones clínicas de las RAMs que los médicos del HIMFG notificaron en el Programa Electrónico de Farmacovigilancia

2.- Describir los medicamentos sospechosos involucrados en las RAMs que los médicos del HIMFG notificaron en el Programa Electrónico de Farmacovigilancia

3.- Describir las características de las RAMs (consecuencia de la RAM, calidad de la información, causalidad, severidad y gravedad) de acuerdo a la NOM-220-SSA1-2012 de Farmacovigilancia que los médicos del HIMFG notificaron en el Programa Electrónico de Farmacovigilancia

VIII. METODOLOGÍA Y PLAN DE ANÁLISIS DE LOS DATOS

Diseño

Análisis secundario de la base de datos del Programa Electrónico de Farmacovigilancia del HIMFG. Debido a que no habrá intervención es un estudio observacional. También corresponde a un estudio descriptivo porque se describirán variables de una población.

Criterios de selección de la población de estudio

Población de estudio: Hasta Diciembre 2017, se encuentran en la base de datos 1,500 notificaciones de RAMs que se han enviado a COFEPRIS

Criterios de Inclusión

- Notificaciones de RAMs que tengan la información de la notificación completa, es decir, que incluya el área que envía el reporte de RAM, la edad del paciente, el sexo, la estatura, el peso, la fecha de inicio de la RAM, la consecuencia de la RAM, la descripción de la RAM, los datos del medicamento sospechoso (dosis, frecuencia, vía de administración, fechas de administración), los datos de los medicamentos que se administraron en presencia de una RAM (dosis, frecuencia, vía de administración, fechas de administración), los diagnósticos, los datos de laboratorio, la gravedad, la severidad, la causalidad, la calidad de la información y los datos del tratamiento de la RAM.

Criterios de Exclusión

- Notificaciones de RAMs duplicadas
- Notificaciones de eventos temporalmente asociados a la vacunación (ETAVs)
- Notificaciones de protocolos de investigación

Muestreo y tamaño de muestra

Tamaño de muestra: Hasta Diciembre 2017, se encuentran en la base de datos 1,500 notificaciones de RAMs que se han enviado a COFEPRIS

Definición de variables

Nombre	
Número de notificaciones de RAMs	**Definición conceptual:** Número de notificaciones de RAMs que los médicos enviaron al Centro Institucional de Farmacovigilancia a través del Sistema Electrónico de Farmacovigilancia del HIMFG<br>**Definición operacional:** Se revisarán y cuantificarán todas las notificaciones de RAMs en la base de datos del Programa Electrónico de Farmacovigilancia del HIMFG que hayan reportado los médicos<br>**Tipo de variable:** Discreta<br>**Unidades de medición:** Sin unidades
Tipo de RAM	**Definición conceptual:** Reacción Adversa a Medicamentos (RAMs) se define como cualquier reacción nociva no intencionada que aparece a dosis normalmente empleadas en el ser humano para la profilaxis, el diagnóstico, el tratamiento o para la modificación de una función fisiológica **[4]**<br>**Definición operacional:** La RAM se clasificó de acuerdo al WHO Adverse Drug Reaction Terminology (WHO-ART) proporcionado por COFEPRIS a través del Sistema SISCE v 1.2.2014<br>**Tipo de variable:** Nominal<br>**Unidades de medición:** Sin Unidades
Medicamento sospechoso de RAM	**Definición conceptual:** Medicamento el cual tiene una relación causal con la RAM de acuerdo al Algoritmo de Naranjo<br>**Definición operacional:** Los principios activos de los medicamentos sospechosos se clasificarán en grupos terapéuticos de acuerdo a The Anatomical Therapeutic Chemical Classification System (ATC/DDD) **[24]**<br>**Tipo de variable:** Nominal<br>**Unidades de medición:** Sin Unidades
Calidad de la información de la notificación de RAM	**Definición conceptual:** Es la exhaustividad e integridad de los datos que contiene la notificación de la RAM **[4]**<br>**Definición operacional:** De acuerdo a la NOM-220-SSA1-2012 cada notificación se clasificó como:<br>Grado 0: Cuando la notificación sólo incluye un paciente identificable, una sospecha de reacción adversa, evento adverso o reacción adversa a un

	medicamento sospechoso y los datos del notificador<br>Grado 1. Cuando además de los datos del Grado 0, se incluyen las fechas de inicio de la sospecha de reacción adversa, evento adverso o reacción adversa y de inicio y término del tratamiento (día, mes y año).<br>Grado 2. Cuando además de los datos del Grado 1, se incluyen denominación genérica y distintiva, posología, vía de administración, motivo de prescripción, consecuencia del evento y datos importantes de la historia clínica.<br>Grado 3. Cuando además de los datos del Grado 2, se incluyen la reaparición de la manifestación clínica consecuente a la re-administración del medicamento (re-administración positiva).<br>**Tipo de variable:** Nominal<br>**Unidades de medición:** Sin unidades
Causalidad de la notificación de RAM	**Definición conceptual:** La valoración de la causalidad es la metodología empleada para estimar la probabilidad de atribuir a un medicamento la RAM observada **[4]**<br>**Definición operacional:** La causalidad de las notificaciones de RAM se evaluó con el Algoritmo de Naranjo, el cual ubica a las RAMs en las siguientes categorías probabilísticas: cierta, probable, posible, dudosa, condicional-inclasificable y no evaluable-inclasificable<br>**Tipo de variable:** Nominal<br>**Unidades de medición:** Sin unidades
Severidad de la RAM	**Definición conceptual:** Se refiere a la intensidad de la manifestación clínica **[4]**<br>**Definición operacional:** De acuerdo a la NOM-220-SSA1-2012 cada notificación se clasificó como:<br>a) Leves. Se presentan con signos y síntomas fácilmente tolerados, no necesitan tratamiento, ni prolongan la hospitalización y no necesariamente requiere de la suspensión del medicamento.<br>b) Moderadas. Interfiere con las actividades habituales (pudiendo provocar bajas laborales o escolares), sin amenazar directamente la vida del paciente. Requiere de tratamiento farmacológico y no necesariamente requiere la suspensión del medicamento causante del evento, reacción o sospecha de reacción adversa.<br>c) Severas. Interfiere con las actividades habituales (pudiendo provocar bajas laborales o escolares). Requiere de tratamiento farmacológico y la

	suspensión del medicamento causante del evento, reacción o sospecha de reacción.<br>**Tipo de variable:** Nominal<br>**Unidades de medición:** Sin unidades
Gravedad de la RAM	**Definición conceptual:** Se refiere al desenlace de la manifestación clínica **[4]**<br>**Definición operacional:** De acuerdo a la NOM-220-SSA1-2012 cada notificación se clasificó como:<br>a) Graves: Cualquier manifestación clínicamente importante que se presenta con la administración de cualquier dosis de un medicamento, y que cumplan los siguientes criterios:<br>- Causan la muerte del paciente<br>- Ponen en peligro la vida del paciente en el momento mismo que se presentan<br>- Hacen necesario hospitalizar o prolongar la estancia hospitalaria<br>- Son causa de invalidez o de incapacidad persistente o significativa<br>- Son causa de alteraciones o malformaciones en el recién nacido<br>b) No Graves. A los eventos, sospechas y reacciones adversas que no cumplan los criterios de gravedad citados anteriormente<br>**Tipo de variable:** Nominal<br>**Unidades de medición:** Sin unidades
Edad de los pacientes	**Definición conceptual:** Tiempo transcurrido desde el nacimiento hasta el día que el paciente presentó la RAM<br>**Definición operacional:** Se calculó con la fecha de nacimiento del paciente y la fecha de inicio de la RAM. Se categorizará de acuerdo a la International Conference on Harmonisation Guideline on Clinical Investigation of Medicinal Products in the Paediatric Population **[25]** considerando los siguientes puntos de corte:<br>- Infantes ≤ 23 meses<br>- Niños 24 a 143 meses (11 años 11 meses)<br>- Adolescentes ≥ 144 meses (12 años)<br>**Tipo de variable:** Nominal<br>**Unidades de medición:** Meses
Sexo de los pacientes	**Definición conceptual:** Condición orgánica que distingue al hombre de la mujer<br>**Definición operacional:** Los médicos registraron en el SISFAR el sexo del

	paciente<br>**Tipo de variable:** Nominal dicotómica<br>**Unidades de medición:** Hombre o mujer
Peso de los pacientes	**Definición conceptual:** Es una medida de la masa corporal total<br>**Definición operacional:** Los médicos registraron en el SISFAR el peso del paciente<br>**Tipo de variable:** Continua<br>**Unidades de medición:** Kilogramos
Talla de los pacientes	**Definición conceptual:** Es la distancia máxima entre la región plantar y el vertex en un plano sagital<br>**Definición operacional:** Los médicos registraron en el SISFAR la talla de cada paciente<br>**Tipo de variable:** Continua<br>**Unidades de medición:** Metros
Número de medicamentos concomitantes	**Definición conceptual:** Número de medicamentos que se administraron al paciente cuando presentó la RAM<br>**Definición operacional:** Los médicos consignaron en el SISFAR los medicamentos que los pacientes recibieron cuando presentó la RAM. Se contabilizarán los medicamentos que cada paciente recibió<br>**Tipo de variable:** Discreta<br>**Unidades de medición:** Sin unidades
Diagnósticos	**Definición conceptual:** Es el procedimiento por el cual se identifica una enfermedad, entidad nosológica, síndrome o cualquier estado patológico o de salud<br>**Definición operacional:** Los médicos registraron en el SISFAR los diagnósticos de los pacientes, los cuales se clasificarán de acuerdo a la CIE-10<br>**Tipo de variable:** Nominal<br>**Unidades de medición:** Sin unidades

Análisis estadístico

Calidad de la información de la base de datos del Sistema de Captura y Evaluación de las Notificaciones de Sospechas de Reacciones Adversas de los Medicamentos y ETAV´S Ver. 1.2 2014 (SISCE v1.2.2014): La base de datos del programa SISCE v1.2.2014 se encuentra en Microsoft Access 2007 por lo que se exportará a Microsoft Office Excel 2007 y SPSS versión 20. Las variables que se exportarán son: número de notificación, departamento que realizó la notificación, iniciales del paciente, fecha de nacimiento, edad, sexo, estatura, peso, fecha de inicio de la RAM, consecuencia del evento, la descripción de la RAM, grupo terapéutico del medicamento sospechoso, información del medicamento sospechoso (principio activo, dosis, vía de administración y fechas de administración), información de los medicamentos concomitantes (principio activo, dosis, vía de administración y fechas de administración), diagnósticos del paciente, información del análisis de la RAM (nombre de la RAM de acuerdo al WHO-ART, gravedad, severidad, causalidad y calidad de la información). Se verificarán los valores extremos (mínimos y máximos) y faltantes de las variables de peso, talla y edad. Posteriormente, para cada paciente se calculará el número de medicamentos concomitantes. Se verificará que todos los casos tengan la información relacionada al nombre del medicamento sospechoso y del análisis de la RAM (nombre de la RAM de acuerdo al WHO-ART, gravedad, severidad, causalidad y calidad de la información).

Análisis descriptivo: Se realizará el análisis descriptivo con medidas de tendencia central y de dispersión como el número de casos (%), media (IC 95%) y mediana (percentiles 25-75) de acuerdo a la naturaleza de cada variable (cuantitativa o cualitativa). Los análisis estadísticos se realizarán con SPSS versión 20.

Procedimientos

Captura de notificaciones de RAMs en el SISFAR:

En el año 2008 el CIHIMFG instaló el SISFAR en las áreas de hospitalización de Oncología, Terapia Intensiva, Neonatología, Nefrología, Medicina Interna, Infectología, Gastroenterología, Cardiología, Reumatología, Urgencias, Cirugía General y de Tórax, Especialidades y Cirugía Cardiovascular del HIMFG para que los médicos capturaran el reporte de las RAMs en línea. El programa SISFAR está basado en los requerimientos de la NOM-220-SSA1-2012 y la

información que los médicos capturaron de cada notificación está distribuida en 7 pestañas, que se describen brevemente a continuación y que se pueden ver en el Anexo 1:

Pestaña 1 - Datos del paciente: Contiene el número de registro, nombre del paciente, dirección, fecha de nacimiento, edad, sexo, estatura, peso y masa corporal.
Pestaña 2 - Reacción Adversa: Contiene datos de alergias a medicamentos, alimentos y otros, fecha de inicio de la reacción, consecuencias, descripción.
Pestaña 3- Medicamento Sospechoso: Contiene el nombre genérico del medicamento, denominación distintiva, laboratorio productor, número de lote, fecha de caducidad, fechas de administración, vía de administración, dosis, motivo de la prescripción.
Pestaña 4 - Farmacoterapia concomitante: Contiene el nombre genérico del medicamento concomitante, dosis, vía de administración, fecha de inicio y término, así como el motivo de la prescripción por medicamento.
Pestaña 5 - Historia clínica: Documenta los datos de diagnóstico, alergias, embarazo, cirugía y datos de laboratorio.
Pestaña 6 - Procedencia de la información: Documenta el nombre del que reportó la reacción, así como su departamento.
Pestaña 7 - Reportes estadísticos: Es una base de datos que puede ser exportada a Excel para el manejo de la información.

Captura y análisis de RAMs en el Sistema de Captura y Evaluación de las Notificaciones de Sospechas de Reacciones Adversas de los Medicamentos y ETAV´S (SISCE v1.2.2014)

Después que los médicos realizaban la captura de su notificación en el SISFAR, estas se recibían en el CIHIMFG para analizarlas y enviarlas electrónicamente al Centro Nacional de Farmacovigilancia a través del programa SISCE v1.2.2014. La información de cada notificación está distribuida en 7 pestañas que se describen a continuación y que se pueden ver en el Anexo 2:

Pestaña 1 Datos del aviso al Centro Nacional de Farmacovigilancia: Número de notificación, fecha de recepción, fecha de captura, área de procedencia de la notificación, origen de la notificación (medicamento, vacuna, protocolo)
Pestaña 2 Datos del paciente: iniciales, fecha de nacimiento, edad, sexo, estatura, peso

Pestaña 3 Datos de la sospecha: fecha de inicio de la RAM, consecuencia del evento, descripción de la RAM

Pestaña 4 Datos de la medicación: Datos del medicamento sospechoso (nombre comercial, lote, grupo terapéutico, caducidad, laboratorio productor, nombre genérico, dosis, frecuencia, vía de administración, fecha de inicio/término y motivo de prescripción), datos de los medicamentos concomitantes (nombre genérico, dosis, frecuencia, vía de administración, fecha de inicio/término y motivo de prescripción)

Pestaña 5 Información de la medicación: Información para el Algoritmo de Naranjo

Pestaña 6 Historia clínica: Diagnósticos y valores de laboratorio relevantes para la RAM

Pestaña 7: Valoración: En esta pestaña un farmacéutico del CIHIMFG realizó el análisis de cada notificación de acuerdo a la NOM-220-SSA1-2012. Se registraron los datos del nombre de la RAM de acuerdo al catálogo WHO Adverse Drug Reaction Terminology (WHO-ART), gravedad, desenlace, intensidad, causalidad, calidad de la información, si la RAM se produjo en el embarazo, si el medicamento sospechoso fue administrado durante la lactancia, si la RAM produjo la hospitalización o amplió la estancia hospitalaria, si la RAM fue por una interacción farmacológica, si el paciente recibió tratamiento y observaciones del analista.

Como se mencionó previamente, luego de que cada notificación fue analizada por un farmacéutico en el CIHIMFG, ésta fue enviada electrónicamente al Centro Nacional de Farmacovigilancia a través del programa SISCE v1.2.2014. El Centro Nacional de Farmacovigilancia envió electrónicamente al CIHIMFG un acuse de recepción de las notificaciones.

Consideraciones Éticas y de Bioseguridad

Ética: El protocolo, cuya metodología es la revisión de una base de datos, corresponde a la categoría de “Investigación sin riesgo” ya que es un método de investigación documental, en el que no se realiza intervención o modificación relacionada con variables fisiológicas, psicológicas o sociales **(Formato Adjunto)**. Por lo anterior, se pone a consideración del Comité de Ética en Investigación la dispensación del Consentimiento Informado por escrito**.** Es importante mencionar que los investigadores no difundirán información que identifique a los pacientes como los nombres, las iniciales o el número de registro institucional.

Bioseguridad: De acuerdo a las instrucciones de llenado del “Formato de Bioseguridad” consideramos que este protocolo “No tiene implicaciones de Bioseguridad” **(Formato Adjunto)**

IX. LIMITACIONES DEL ESTUDIO

La principal limitación del estudio es el subreporte de RAMs

X. BIBLIOGRAFÍA

1. Rodríguez-Betancourt J, García-Vigil J, Giral-Barnés C, Hernández-Santillán D, Jasso-Gutiérrez L. Farmacovigilancia I. El inicio. Rev Med Inst Mex Seguro Soc. 2004; 42 (4): 327-329
2. Uppsala Monitoring Centre. https://www.who-umc.org/global-pharmacovigilance/members/. Accesed 8 January 2018
3. Uppsala Monitoring Centre. https://www.who-umc.org/global-pharmacovigilance/global-pharmacovigilance/glossary/. Accesed 8 January 2018
4. Diario Oficial de la Federación. Norma Oficial Mexicana NOM-220-SSA1-2012, Instalación y operación de la Farmacovigilancia. http://dof.gob.mx/nota_detalle.php?codigo=5284236&fecha=07/01/2013. Accesed 8 January 2018
5. Lindell-Osuagwu L, Korhonen MJ, Saano S, Helin-Tanninen M, Naaranlahti T, Kokki H. Off-label and unlicensed drug prescribing in three paediatric wards in Finland and review of the international literature. J Clin Pharm Ther. 2009;34(3):277-287

6. Meador KJ, Baker GA, Browning N, Clayton-Smith J, Combs-Cantrell DT, Cohen M, et al. Effects of breastfeeding in children of women taking antiepileptic drugs. Neurology. 2010;75(22):1954-60

7. Meador KJ, Baker GA, Browning N, Cohen MJ, Bromley RL, Clayton-Smith J, et al. Fetal antiepileptic drug exposure and cognitive outcomes at age 6 years (NEAD study): a prospective observational study. Lancet Neurol. 2013;12(3):244-52

8. Kearns GL, Abdel-Rahman SM, Alander SW, Blowey DL, Leeder JS, Kauffman 230 RE. Developmental pharmacology--drug disposition, action, and therapy in infants and children. N Engl J Med. 2003;349(12):1157-1167

9. Khan L. Comparative epidemiology of hospital-acquired adverse drug reactions in adults and children and their impact on cost and hospital stay--a systematic review. Eur J Clin Pharmacol. 2013;69:1985-96

10. Smyth R, Gargon E, Kirkham J, et al. Adverse drug reactions in children--a systematic review. PLoS One. 2012;7:e24061

11. Telechea H, Speranza N, Lucas L, Giachetto G, Nanni L, Menchaca A. Adverse drug reactions in a paediatric intensive care unit. Farm Hosp. 2012;36:403-9

12. Gill AM, Leach HJ, Hughes J, Barker C, Nunn AJ, Choonara I. Adverse drug reactions in a paediatric intensive care unit. Acta Paediatr. 1995; 84:438-41

13. Du W, Tutag Lehr V, Caverly M, Kelm L, Reeves J, Lieh-Lai M. Incidence and costs of adverse drug reactions in a tertiary care pediatric intensive care unit. J Clin Pharmacol. 2013;53:567-73

14. Zed PJ, Black KJ, Fitzpatrick EA, Ackroyd-Stolarz S, Murphy NG, Curran JA, et al. Medication-related emergency department visits in pediatrics: a prospective observational study. Pediatrics. 2015;135:435-43

15. Star K, Norén G, Nordin K, Edwards I. Suspected adverse drug reactions reported for children worldwide: an exploratory study using VigiBase. Drug Saf. 2011;34:415-28

16. Finkelstein Y, Soon GS, Acuna P, George M, Pope E, Ito S, et al. Recurrence and outcomes of Stevens-Johnson syndrome and toxic epidermal necrolysis in children. Pediatrics. 2011;128(4):723-8

17. Gallagher RM, Mason JR, Bird KA, Kirkham JJ, Peak M, Williamson PR, et al. Adverse drug reactions causing admission to a paediatric hospital. PLoS One. 2012;7(12):e50127

18. Oshikoya KA, Chukwura H, Njokanma OF, Senbanjo IO, Ojo I. Incidence and cost estimate of treating pediatric adverse drug reactions in Lagos, Nigeria. Sao Paulo Med J. 2011;129(3):153-64

19. Thürmann P. Methods and systems to detect adverse drug reactions in hospitals. Drug Saf. 2001;24:961-8

20. Strom BL, Kimmel SE, Hennessy S, eds. Pharmacoepidemiology. West Sussex Inglaterra:Wiley-Blackwell; 2012

21. Hazell L, Shakir SA. Under-reporting of adverse drug reactions: a systematic review. Drug Saf. 2006;29:385-96

22. Morales Ríos O, Jasso Gutiérrez L, Talavera JO, Téllez-Rojo MM, Olivar López V, Garduño Espinosa J, et al. A comprehensive intervention for adverse drug reactions identification and reporting in a Pediatric Emergency Department. Int J Clin Pharm. 2016;38(1):80-7.

23. Jasso Gutiérrez L, Ovando Hernández R, Castellanos Solís EC, Escorza Peña J, Santos Preciado JI. Diseño e implantación de un programa electrónico de Farmacovigilancia con captura en línea en el Hospital Infantil de México Federico Gómez. Bol Med Hosp Infant Mex. 2009;66:51-9

24. ATC/DDD Index 2018. https://www.whocc.no/atc_ddd_index/. Accesed 8 January 2018

25. European Medicines Agency. ICH Topic E 11. Clinical Investigation of Medicinal Products in the Paediatric Population. http://www.ema.europa.eu/docs/en_GB/document_library/Scientific_guideline/2009/09/WC500002926.pdf. Accesed 17 September 2015

XI. ANEXOS

Anexo 1.- Formato electrónico de captura de RAMs (SISFAR)

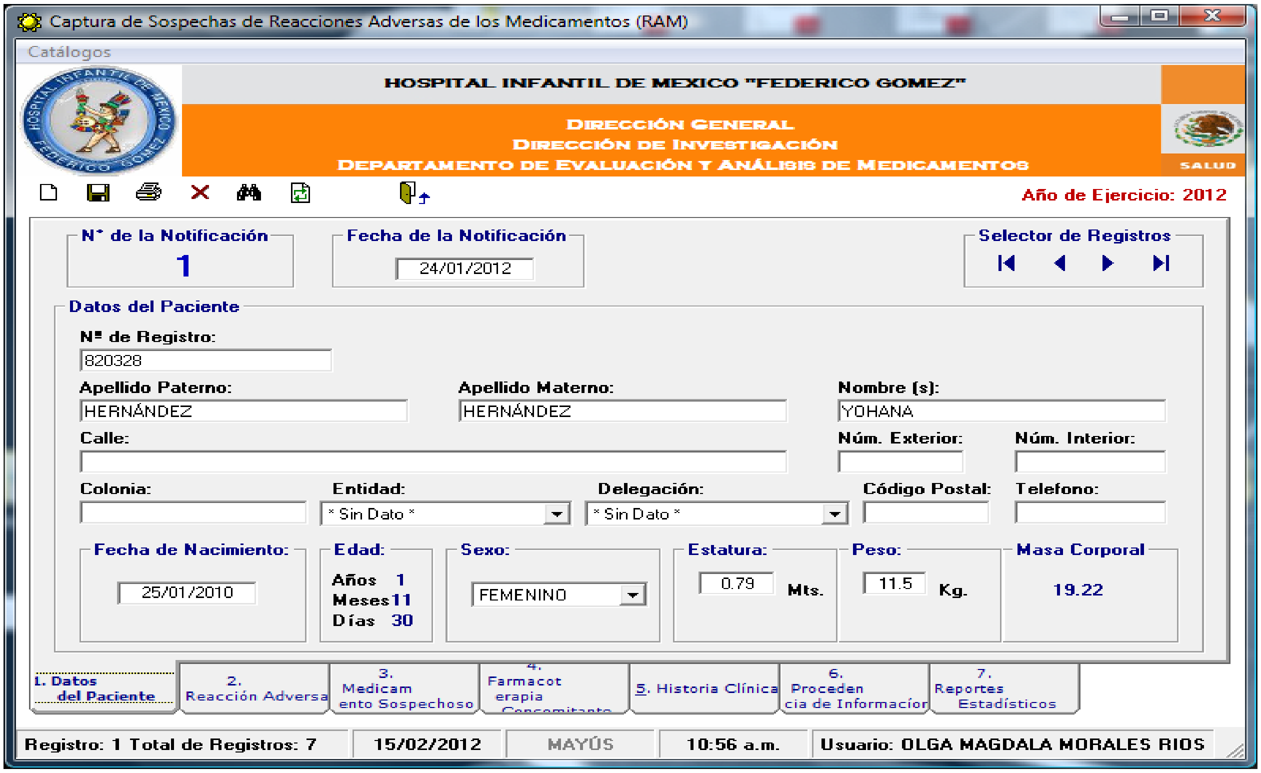

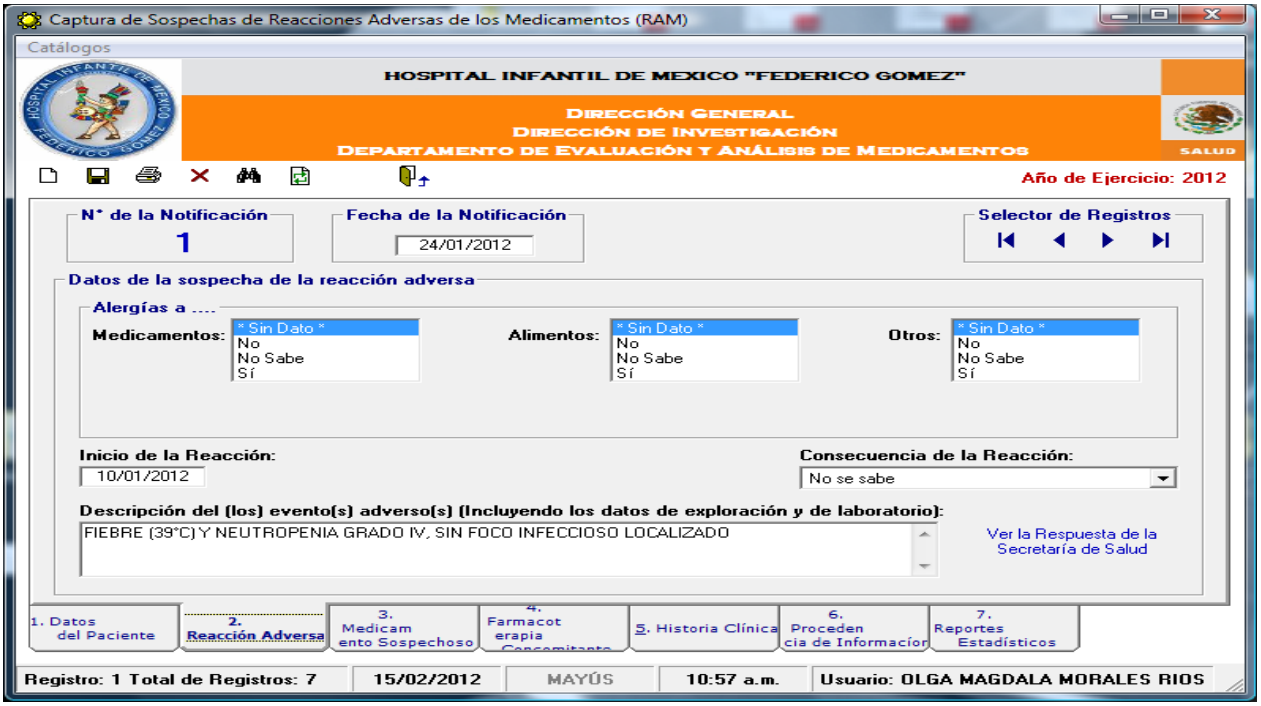
Captura de Sospechas de Reacciones Adversas de los Medicamentos (RAM)
Catálogos
HOSPITAL INFANTIL DE MEXICO "FEDERICO GOMEZ"
DIRECCIÓN GENERAL
DIRECCIÓN DE INVESTIGACIÓN
DEPARTAMENTO DE EVALUACIÓN Y ANÁLISIS DE MEDICAMENTOS
SALUD
Año de Ejercicio: 2012
N° de la Notificación
1
Fecha de la Notificación
24/01/2012
Selector de Registros
Datos de la sospecha de la reacción adversa
Alergías a ....
Medicamentos:
* Sin Dato *
No
No Sabe
Sí
Alimentos:
* Sin Dato *
No
No Sabe
Sí
Otros:
* Sin Dato *
No
No Sabe
Sí
Inicio de la Reacción:
10/01/2012
Consecuencia de la Reacción:
No se sabe
Descripción del (los) evento(s) adverso(s) (Incluyendo los datos de exploración y de laboratorio):
FIEBRE (39°C) Y NEUTROPENIA GRADO IV, SIN FOCO INFECCIOSO LOCALIZADO
Ver la Respuesta de la Secretaría de Salud
1. Datos del Paciente
2. Reacción Adversa
3. Medicamento Sospechoso
4. Farmacoterapia
5. Historia Clínica
6. Procedencia de Información
7. Reportes Estadísticos
Registro: 1 Total de Registros: 7
15/02/2012
MAYÚS
10:57 a.m.
Usuario: OLGA MAGDALA MORALES RIOS

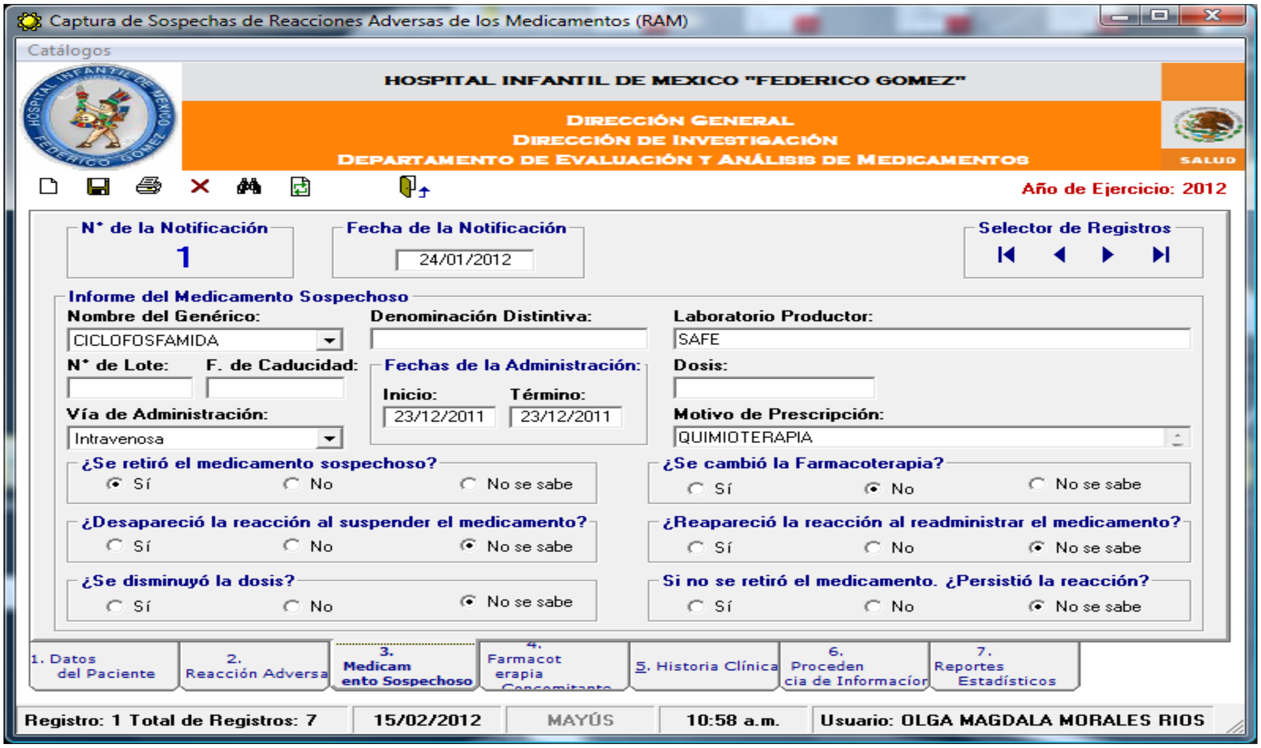
Captura de Sospechas de Reacciones Adversas de los Medicamentos (RAM)
Catálogos
HOSPITAL INFANTIL DE MEXICO "FEDERICO GOMEZ"
DIRECCIÓN GENERAL
DIRECCIÓN DE INVESTIGACIÓN
DEPARTAMENTO DE EVALUACIÓN Y ANÁLISIS DE MEDICAMENTOS
SALUD
Año de Ejercicio: 2012
N° de la Notificación
1
Fecha de la Notificación
24/01/2012
Selector de Registros
Informe del Medicamento Sospechoso
Nombre del Genérico:
CICLOFOSFAMIDA
Denominación Distintiva:
Laboratorio Productor:
SAFE
N° de Lote:
F. de Caducidad:
Fechas de la Administración:
Inicio:
23/12/2011
Término:
23/12/2011
Dosis:
Vía de Administración:
Intravenosa
Motivo de Prescripción:
QUIMIOTERAPIA
¿Se retiró el medicamento sospechoso?
Sí
No
No se sabe
¿Se cambió la Farmacoterapia?
Sí
No
No se sabe
¿Desapareció la reacción al suspender el medicamento?
Sí
No
No se sabe
¿Reapareció la reacción al readministrar el medicamento?
Sí
No
No se sabe
¿Se disminuyó la dosis?
Sí
No
No se sabe
Si no se retiró el medicamento. ¿Persistió la reacción?
Sí
No
No se sabe
1. Datos del Paciente
2. Reacción Adversa
3. Medicamento Sospechoso
4. Farmacoterapia
5. Historia Clínica
6. Procedencia de Información
7. Reportes Estadísticos
Registro: 1 Total de Registros: 7
15/02/2012
MAYÚS
10:58 a.m.
Usuario: OLGA MAGDALA MORALES RIOS

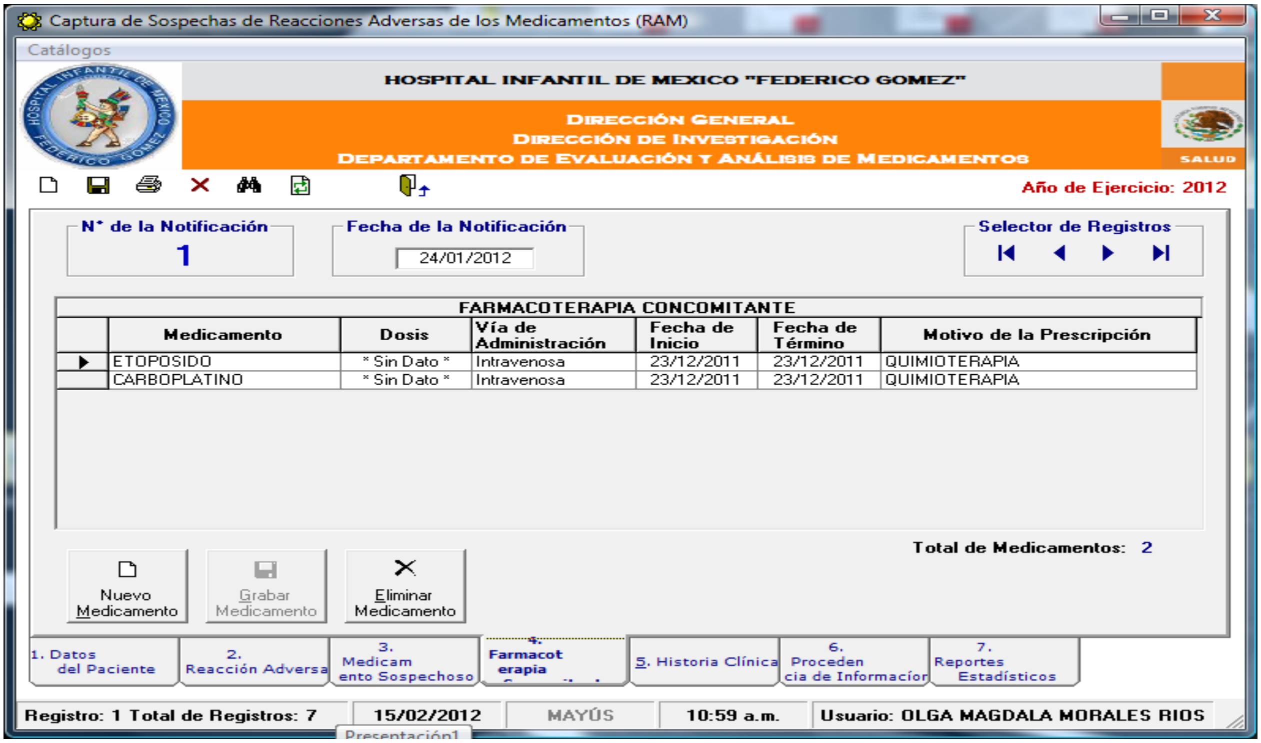
Captura de Sospechas de Reacciones Adversas de los Medicamentos (RAM)
Catálogos
HOSPITAL INFANTIL DE MEXICO "FEDERICO GOMEZ"
DIRECCIÓN GENERAL
DIRECCIÓN DE INVESTIGACIÓN
DEPARTAMENTO DE EVALUACIÓN Y ANÁLISIS DE MEDICAMENTOS
SALUD
Año de Ejercicio: 2012
N° de la Notificación
1
Fecha de la Notificación
24/01/2012
Selector de Registros
FARMACOTERAPIA CONCOMITANTE
Medicamento
Dosis
Vía de Administración
Fecha de Inicio
Fecha de Término
Motivo de la Prescripción
ETOPOSIDO
* Sin Dato *
Intravenosa
23/12/2011
23/12/2011
QUIMIOTERAPIA
CARBOPLATINO
* Sin Dato *
Intravenosa
23/12/2011
23/12/2011
QUIMIOTERAPIA
Total de Medicamentos: 2
Nuevo Medicamento
Grabar Medicamento
Eliminar Medicamento
1. Datos del Paciente
2. Reacción Adversa
3. Medicamento Sospechoso
4. Farmacoterapia
5. Historia Clínica
6. Procedencia de Información
7. Reportes Estadísticos
Registro: 1 Total de Registros: 7
15/02/2012
MAYÚS
10:59 a.m.
Usuario: OLGA MAGDALA MORALES RIOS
Presentación1

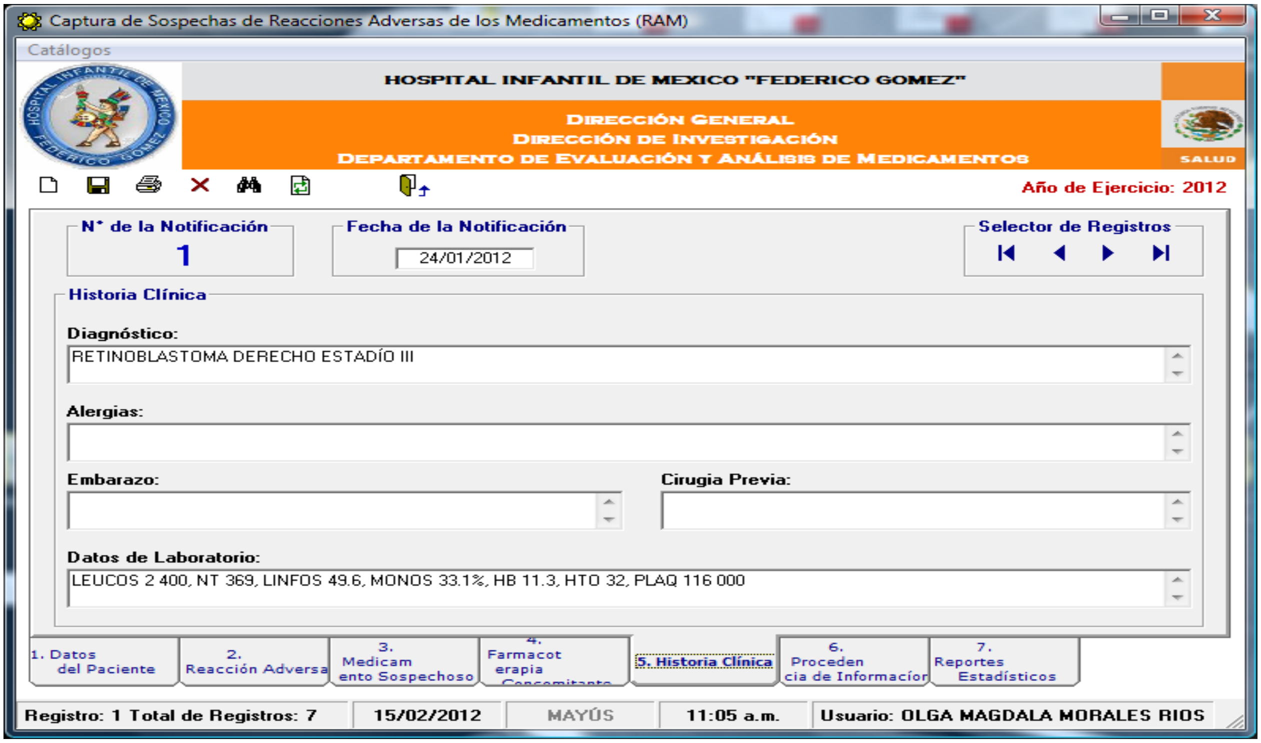
Captura de Sospechas de Reacciones Adversas de los Medicamentos (RAM)
Catálogos
HOSPITAL INFANTIL DE MEXICO "FEDERICO GOMEZ"
DIRECCIÓN GENERAL
DIRECCIÓN DE INVESTIGACIÓN
DEPARTAMENTO DE EVALUACIÓN Y ANÁLISIS DE MEDICAMENTOS
SALUD
Año de Ejercicio: 2012
N° de la Notificación
1
Fecha de la Notificación
24/01/2012
Selector de Registros
Historia Clínica
Diagnóstico:
RETINOBLASTOMA DERECHO ESTADÍO III
Alergias:
Embarazo:
Cirugia Previa:
Datos de Laboratorio:
LEUCOS 2 400, NT 369, LINFOS 49.6, MONOS 33.1%, HB 11.3, HTO 32, PLAQ 116 000
1. Datos del Paciente
2. Reacción Adversa
3. Medicamento Sospechoso
4. Farmacoterapia
5. Historia Clínica
6. Procedencia de Información
7. Reportes Estadísticos
Registro: 1 Total de Registros: 7
15/02/2012
MAYÚS
11:05 a.m.
Usuario: OLGA MAGDALA MORALES RIOS

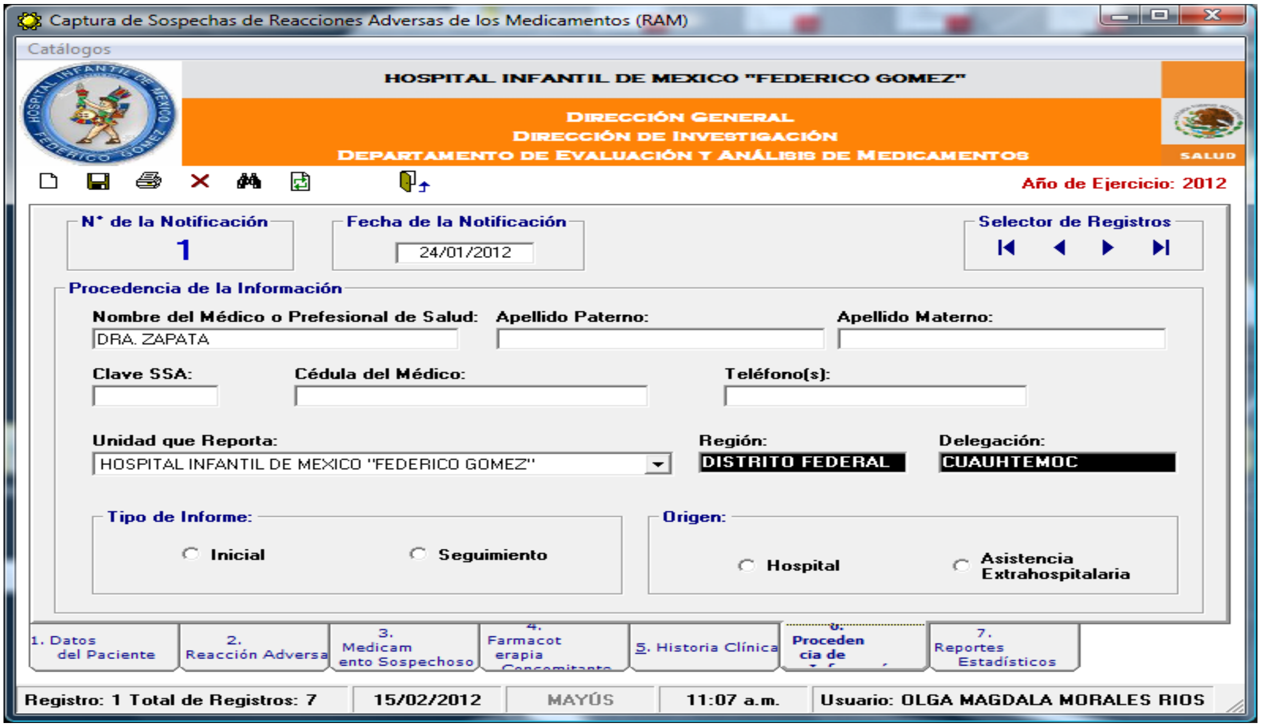
Captura de Sospechas de Reacciones Adversas de los Medicamentos (RAM)
Catálogos
HOSPITAL INFANTIL DE MEXICO "FEDERICO GOMEZ"
DIRECCIÓN GENERAL
DIRECCIÓN DE INVESTIGACIÓN
DEPARTAMENTO DE EVALUACIÓN Y ANÁLISIS DE MEDICAMENTOS
SALUD
Año de Ejercicio: 2012
N° de la Notificación
1
Fecha de la Notificación
24/01/2012
Selector de Registros
Procedencia de la Información
Nombre del Médico o Prefesional de Salud:
DRA. ZAPATA
Apellido Paterno:
Apellido Materno:
Clave SSA:
Cédula del Médico:
Teléfono(s):
Unidad que Reporta:
HOSPITAL INFANTIL DE MEXICO "FEDERICO GOMEZ"
Región:
DISTRITO FEDERAL
Delegación:
CUAUHTEMOC
Tipo de Informe:
Inicial
Seguimiento
Origen:
Hospital
Asistencia Extrahospitalaria
1. Datos del Paciente
2. Reacción Adversa
3. Medicamento Sospechoso
4. Farmacoterapia
5. Historia Clínica
7. Reportes Estadísticos
Registro: 1 Total de Registros: 7
15/02/2012
MAYÚS
11:07 a.m.
Usuario: OLGA MAGDALA MORALES RIOS

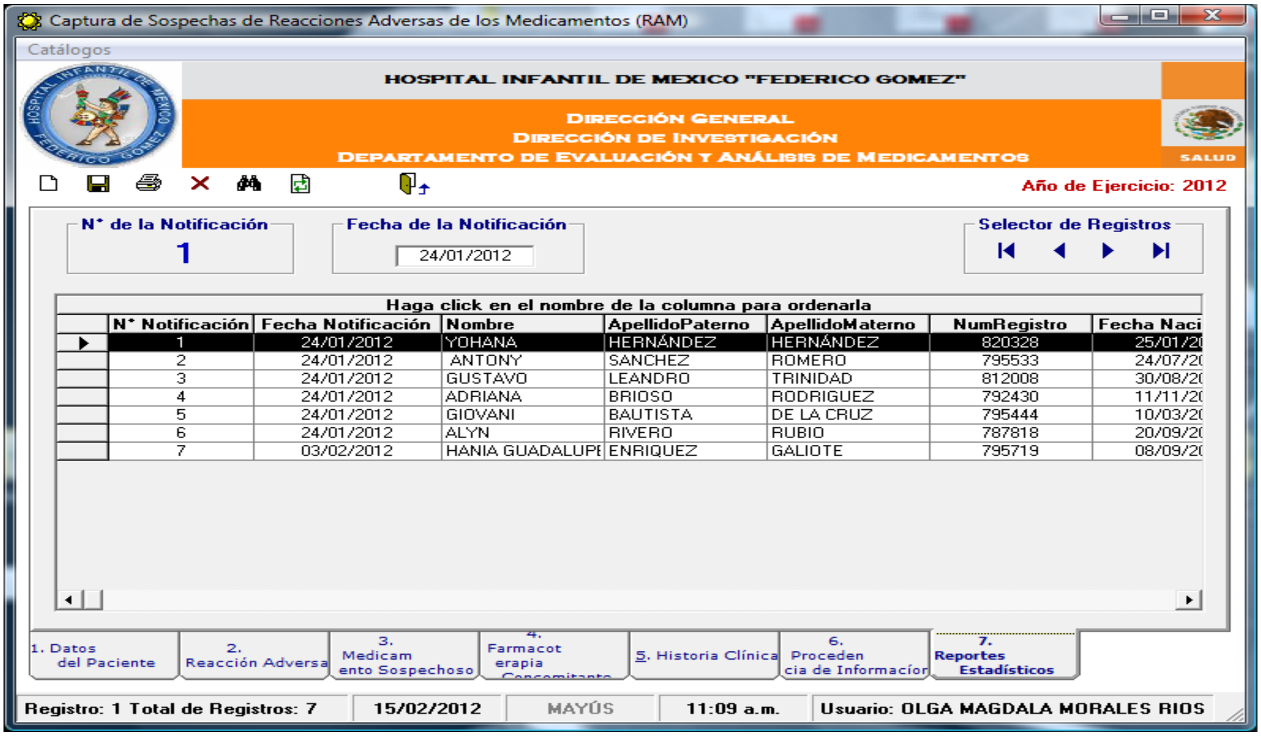
Captura de Sospechas de Reacciones Adversas de los Medicamentos (RAM)
Catálogos
HOSPITAL INFANTIL DE MEXICO "FEDERICO GOMEZ"
DIRECCIÓN GENERAL
DIRECCIÓN DE INVESTIGACIÓN
DEPARTAMENTO DE EVALUACIÓN Y ANÁLISIS DE MEDICAMENTOS
SALUD
Año de Ejercicio: 2012
N° de la Notificación
1
Fecha de la Notificación
24/01/2012
Selector de Registros
Haga click en el nombre de la columna para ordenarla
N° Notificación | Fecha Notificación | Nombre | ApellidoPaterno | ApellidoMaterno | NumRegistro | Fecha Naci
1 | 24/01/2012 | YOHANA | HERNÁNDEZ | HERNÁNDEZ | 820328 | 25/01/20
2 | 24/01/2012 | ANTONY | SANCHEZ | ROMERO | 795533 | 24/07/20
3 | 24/01/2012 | GUSTAVO | LEANDRO | TRINIDAD | 812008 | 30/08/20
4 | 24/01/2012 | ADRIANA | BRIOSO | RODRIGUEZ | 792430 | 11/11/20
5 | 24/01/2012 | GIOVANI | BAUTISTA | DE LA CRUZ | 795444 | 10/03/20
6 | 24/01/2012 | ALYN | RIVERO | RUBIO | 787818 | 20/09/20
7 | 03/02/2012 | HANIA GUADALUPE | ENRIQUEZ | GALIOTE | 795719 | 08/09/20
1. Datos del Paciente
2. Reacción Adversa
3. Medicamento Sospechoso
4. Farmacoterapia
5. Historia Clínica
6. Procedencia de Información
7. Reportes Estadísticos
Registro: 1 Total de Registros: 7
15/02/2012
MAYÚS
11:09 a.m.
Usuario: OLGA MAGDALA MORALES RIOS

Anexo 2.- Sistema de Captura y Evaluación de las Notificaciones de Sospechas de Reacciones Adversas de los Medicamentos y ETAV´S (SISCE v1.2.2014)

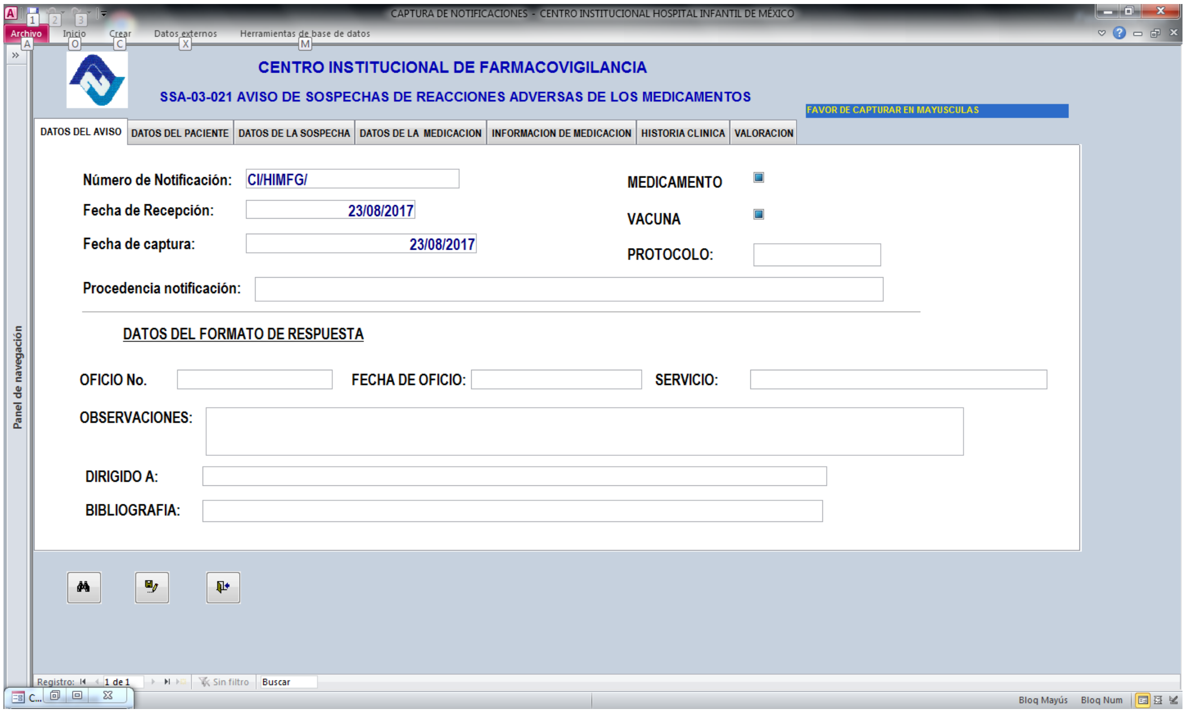

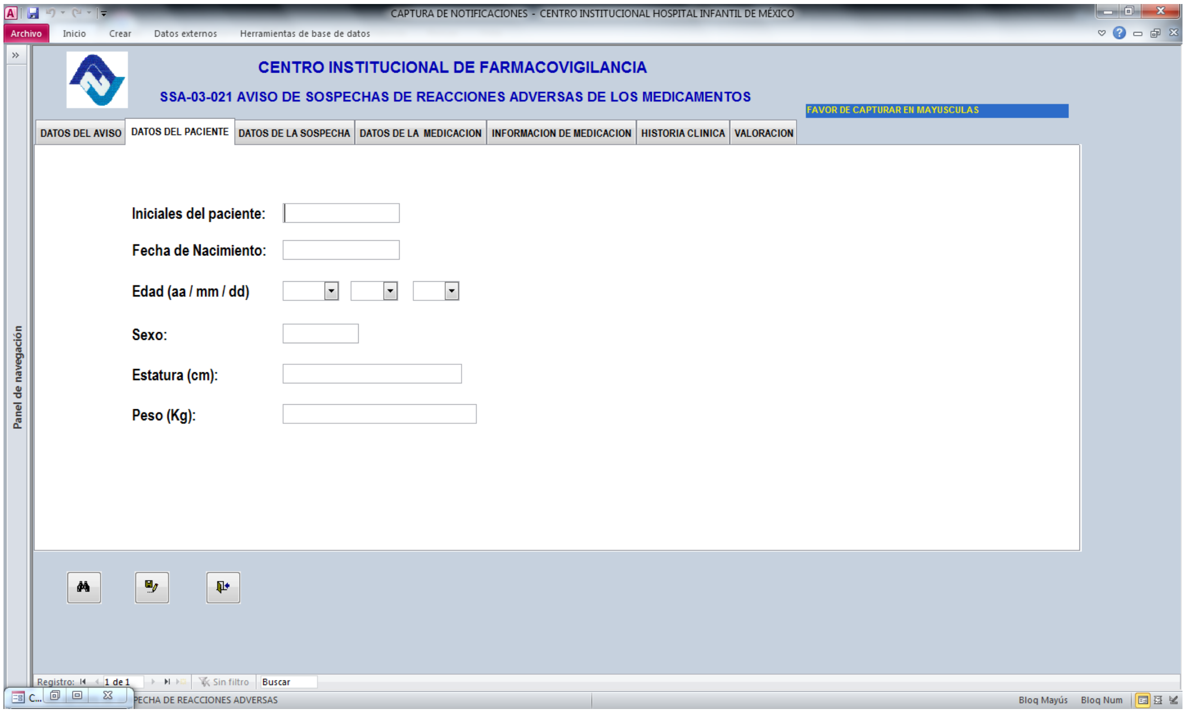

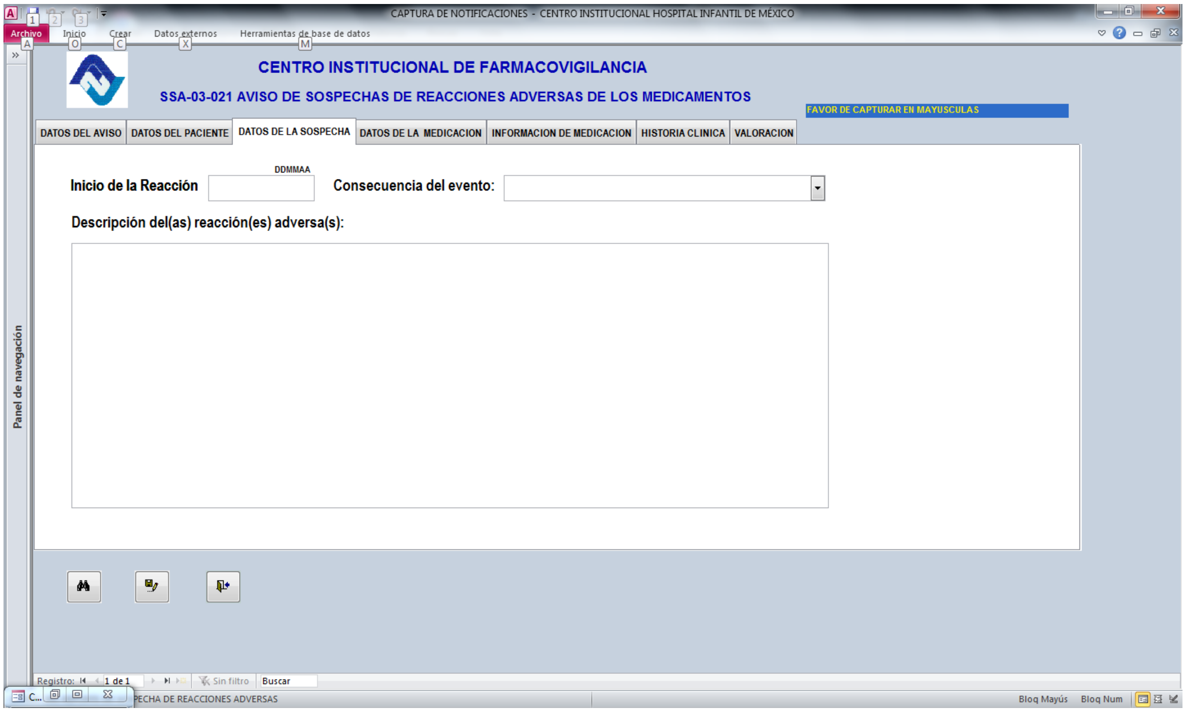
CAPTURA DE NOTIFICACIONES - CENTRO INSTITUCIONAL HOSPITAL INFANTIL DE MÉXICO
Archivo
Inicio
Crear
Datos externos
Herramientas de base de datos
CENTRO INSTITUCIONAL DE FARMACOVIGILANCIA
SSA-03-021 AVISO DE SOSPECHAS DE REACCIONES ADVERSAS DE LOS MEDICAMENTOS
FAVOR DE CAPTURAR EN MAYUSCULAS
DATOS DEL AVISO
DATOS DEL PACIENTE
DATOS DE LA SOSPECHA
DATOS DE LA MEDICACION
INFORMACION DE MEDICACION
HISTORIA CLINICA
VALORACION
DDMMAA
Inicio de la Reacción
Consecuencia del evento:
Descripción del(as) reacción(es) adversa(s):
Panel de navegación
Registro: 1 de 1
Sin filtro
Buscar
Bloq Mayús
Bloq Num

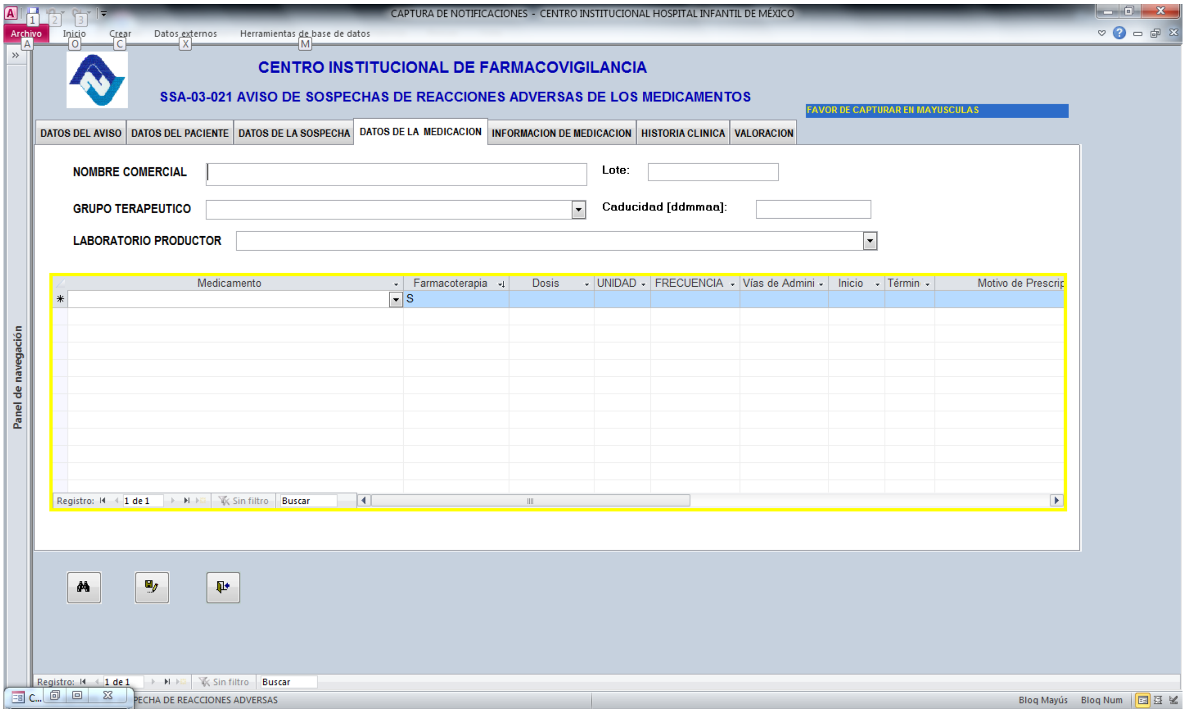
CAPTURA DE NOTIFICACIONES - CENTRO INSTITUCIONAL HOSPITAL INFANTIL DE MÉXICO
Archivo
Inicio
Crear
Datos externos
Herramientas de base de datos
CENTRO INSTITUCIONAL DE FARMACOVIGILANCIA
SSA-03-021 AVISO DE SOSPECHAS DE REACCIONES ADVERSAS DE LOS MEDICAMENTOS
FAVOR DE CAPTURAR EN MAYUSCULAS
DATOS DEL AVISO
DATOS DEL PACIENTE
DATOS DE LA SOSPECHA
DATOS DE LA MEDICACION
INFORMACION DE MEDICACION
HISTORIA CLINICA
VALORACION
NOMBRE COMERCIAL
Lote:
GRUPO TERAPEUTICO
Caducidad [ddmmaa]:
LABORATORIO PRODUCTOR
Medicamento
Farmacoterapia
Dosis
UNIDAD
FRECUENCIA
Vías de Admini
Inicio
Térmi
Motivo de Prescrip
S
Registro: 1 de 1
Sin filtro
Buscar
Panel de navegación
Bloq Mayús
Bloq Num

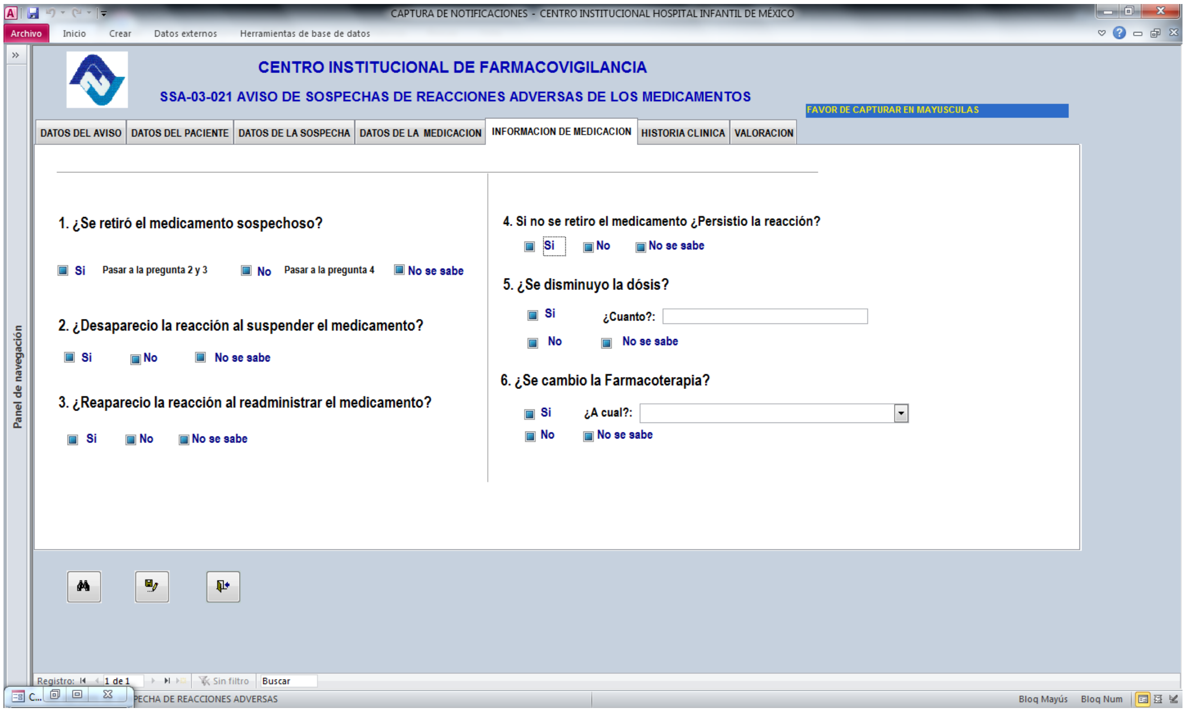
CAPTURA DE NOTIFICACIONES - CENTRO INSTITUCIONAL HOSPITAL INFANTIL DE MÉXICO
Archivo
Inicio
Crear
Datos externos
Herramientas de base de datos
CENTRO INSTITUCIONAL DE FARMACOVIGILANCIA
SSA-03-021 AVISO DE SOSPECHAS DE REACCIONES ADVERSAS DE LOS MEDICAMENTOS
FAVOR DE CAPTURAR EN MAYUSCULAS
DATOS DEL AVISO
DATOS DEL PACIENTE
DATOS DE LA SOSPECHA
DATOS DE LA MEDICACION
INFORMACION DE MEDICACION
HISTORIA CLINICA
VALORACION
1. ¿Se retiró el medicamento sospechoso?
Si
Pasar a la pregunta 2 y 3
No
Pasar a la pregunta 4
No se sabe
2. ¿Desaparecio la reacción al suspender el medicamento?
Si
No
No se sabe
3. ¿Reaparecio la reacción al readministrar el medicamento?
Si
No
No se sabe
4. Si no se retiro el medicamento ¿Persistio la reacción?
Si
No
No se sabe
5. ¿Se disminuyo la dósis?
Si
¿Cuanto?:
No
No se sabe
6. ¿Se cambio la Farmacoterapia?
Si
¿A cual?:
No
No se sabe
Panel de navegación
Registro: 1 de 1
Sin filtro
Buscar
PECHA DE REACCIONES ADVERSAS
Bloq Mayús
Bloq Num

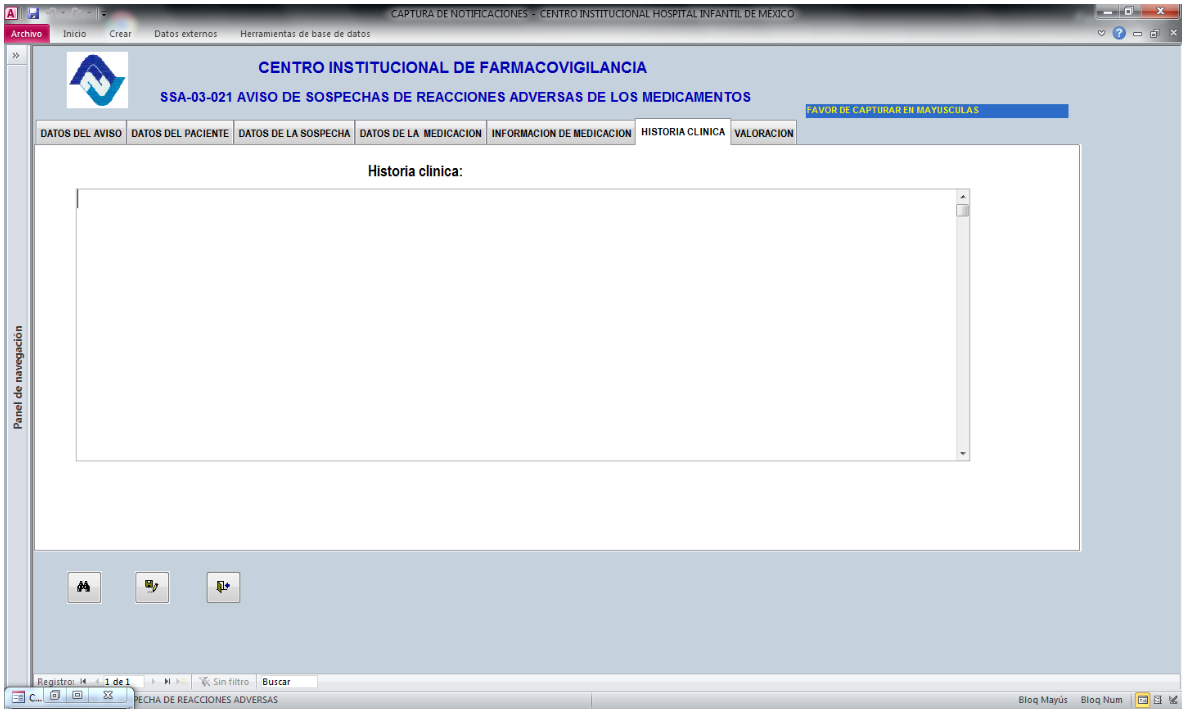
CAPTURA DE NOTIFICACIONES - CENTRO INSTITUCIONAL HOSPITAL INFANTIL DE MÉXICO
Archivo
Inicio
Crear
Datos externos
Herramientas de base de datos
CENTRO INSTITUCIONAL DE FARMACOVIGILANCIA
SSA-03-021 AVISO DE SOSPECHAS DE REACCIONES ADVERSAS DE LOS MEDICAMENTOS
FAVOR DE CAPTURAR EN MAYUSCULAS
DATOS DEL AVISO
DATOS DEL PACIENTE
DATOS DE LA SOSPECHA
DATOS DE LA MEDICACION
INFORMACION DE MEDICACION
HISTORIA CLINICA
VALORACION
Historia clinica:
Panel de navegación
Registro: 1 de 1
Sin filtro
Buscar
PECHA DE REACCIONES ADVERSAS
Bloq Mayús
Bloq Num

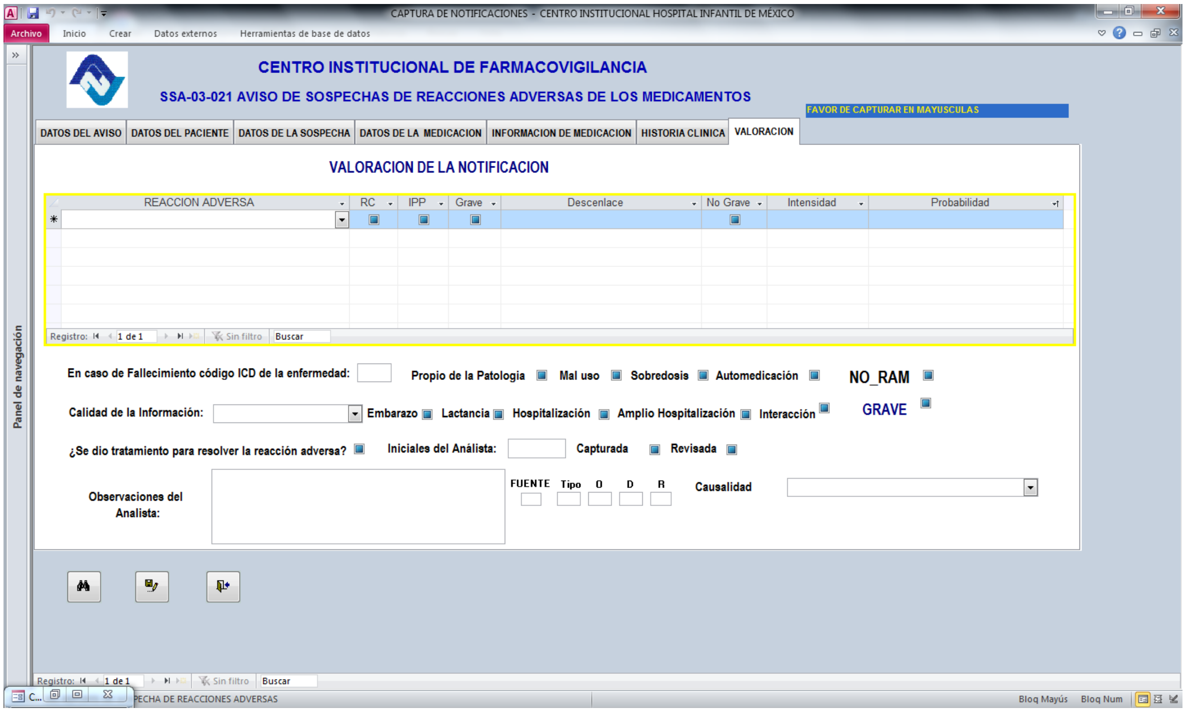
CAPTURA DE NOTIFICACIONES - CENTRO INSTITUCIONAL HOSPITAL INFANTIL DE MÉXICO
Archivo
Inicio
Crear
Datos externos
Herramientas de base de datos
CENTRO INSTITUCIONAL DE FARMACOVIGILANCIA
SSA-03-021 AVISO DE SOSPECHAS DE REACCIONES ADVERSAS DE LOS MEDICAMENTOS
FAVOR DE CAPTURAR EN MAYUSCULAS
DATOS DEL AVISO
DATOS DEL PACIENTE
DATOS DE LA SOSPECHA
DATOS DE LA MEDICACION
INFORMACION DE MEDICACION
HISTORIA CLINICA
VALORACION
VALORACION DE LA NOTIFICACION
REACCION ADVERSA
RC
IPP
Grave
Descenlace
No Grave
Intensidad
Probabilidad
Registro: 1 de 1
Sin filtro
Buscar
En caso de Fallecimiento código ICD de la enfermedad:
Propio de la Patologia
Mal uso
Sobredosis
Automedicación
NO_RAM
Calidad de la Información:
Embarazo
Lactancia
Hospitalización
Amplio Hospitalización
Interacción
GRAVE
¿Se dio tratamiento para resolver la reacción adversa?
Iniciales del Análista:
Capturada
Revisada
Observaciones del Analista:
FUENTE
Tipo
O
D
R
Causalidad
Panel de navegación
Bloq Mayús
Bloq Num
